# The B Cell Antigen Receptor and Overexpression of *MYC* Can Cooperate in the Genesis of B Cell Lymphomas

**DOI:** 10.1371/journal.pbio.0060152

**Published:** 2008-06-24

**Authors:** Yosef Refaeli, Ryan M Young, Brian C Turner, Jennifer Duda, Kenneth A Field, J. Michael Bishop

**Affiliations:** 1 The George W. Hooper Foundation, University of California, San Francisco, San Francisco, California, United States of America; 2 Department of Microbiology and Immunology, University of California, San Francisco, San Francisco, California, United States of America; 3 Department of Pediatrics, Program in Cell Biology, National Jewish Medical and Research Center, Denver, Colorado, United States of America; University of Wisconsin, Madison, United States of America

## Abstract

A variety of circumstantial evidence from humans has implicated the B cell antigen receptor (BCR) in the genesis of B cell lymphomas. We generated mouse models designed to test this possibility directly, and we found that both the constitutive and antigen-stimulated state of a clonal BCR affected the rate and outcome of lymphomagenesis initiated by the proto-oncogene *MYC*. The tumors that arose in the presence of constitutive BCR differed from those initiated by *MYC* alone and resembled chronic B cell lymphocytic leukemia/lymphoma (B-CLL), whereas those that arose in response to antigen stimulation resembled large B-cell lymphomas, particularly Burkitt lymphoma (BL). We linked the genesis of the BL-like tumors to antigen stimulus in three ways. First, in reconstruction experiments, stimulation of B cells by an autoantigen in the presence of overexpressed *MYC* gave rise to BL-like tumors that were, in turn, dependent on both *MYC* and the antigen for survival and proliferation. Second, genetic disruption of the pathway that mediates signaling from the BCR promptly killed cells of the BL-like tumors as well as the tumors resembling B-CLL. And third, growth of the murine BL could be inhibited by any of three distinctive immunosuppressants, in accord with the dependence of the tumors on antigen-induced signaling. Together, our results provide direct evidence that antigenic stimulation can participate in lymphomagenesis, point to a potential role for the constitutive BCR as well, and sustain the view that the constitutive BCR gives rise to signals different from those elicited by antigen. The mouse models described here should be useful in exploring further the pathogenesis of lymphomas, and in preclinical testing of new therapeutics.

## Author Summary

It has long been suspected that the malignant proliferation of B lymphocytes known as lymphomas might represent a perversion of how the cells normally respond to antigen. In particular, the molecular receptor on the surface of the cells that signals the presence of antigen might be abnormally active in lymphomas. We have tested this hypothesis by engineering the genome of mice so that virtually all of the B cells are commandeered by a single version of the surface receptor, then stimulated that receptor with the molecule it is designed to recognize. Our results indicate that both the unstimulated and stimulated states of the receptor can cooperate with an oncogene known as *MYC* in the genesis of lymphomas. But the two states of the receptor give rise to different forms of lymphoma. In particular, the stimulated form cooperates with *MYC* to produce a disease that closely resembles Burkitt lymphoma. These results illuminate the mechanisms that are responsible for lymphomas and could inform the development of new strategies to treat the disease.

## Abstract 3

A series of genetically engineered mice were used to substantiate a long-standing speculation that chronic immune-stimulus may be involved in the genesis of certain lymphomas, illuminating the pathogenesis of B cell lymphomas and suggesting new strategies to treat several forms of this malignancy, including Burkitt lymphoma.

## Introduction

Malignancies affecting the B cell lineage comprise the vast majority of human lymphomas [1]. There are at least 15 different types of B cell lymphomas (BCLs), differing in clinical behavior, biological phenotype, pathogenesis, and response to treatment. Irrespective of their type, however, most BCLs share two features: chromosomal translocations that involve an immunoglobulin gene and one or another proto-oncogene [2], and expression of a B cell antigen receptor (BCR). Chromosomal translocations have long been considered crucial to the pathogenesis of the tumors. But there is now increasing evidence that signaling from the BCR may be a coconspirator in that pathogenesis (for a review, see [3]).

A BCR is expressed on normal B cells throughout the course of their development, and this expression appears to be essential for survival of the cells [4]. There is controversy, however, about whether the life-sustaining signal from the BCR is autogenous in nature or arises from antigenic stimulus [5]. The BCR expressed by BCLs is also apparently required for survival of the tumor cells and may drive cellular proliferation [6].

More than 40 years ago, Damashek and Schwartz proposed that antigenic stimulus might contribute to the genesis of BCLs in the context of autoimmune disease [7]. In the interim, circumstantial evidence has mounted to support a role for antigen stimulation in diverse forms of lymphomagenesis. For example, in some instances, the structure of the BCR on BCLs shows evidence of having been subjected to antigen selection [8–14], and may even bind a known antigen—either a protein encoded by a virus suspected of being an etiological agent, or an autoantigen [15,16].

We sought to test directly the role of the BCR in the genesis of BCLs by reconstruction in mouse models. We used a series of transgenic mice that allowed cooperation between either the constitutive or antigen-activated BCR with the proto-oncogene *MYC*, the activation of which by chromosomal translocation has been implicated in the genesis of human diffuse large B cell lymphoma and Burkitt lymphoma (BL) [17–19]. We derived these models from two strains of mice that express transgenes of *MYC* in the lymphoid lineage. In one strain (Eμ-*MYC*), transcription of the transgene is driven by the control element for the immunoglobulin heavy chain gene [20]; in the other strain (MMTV-rtTA/TRE-*MYC*), the *MYC* transgene is also transcribed in the B cell lineage, but the transcription is governed by a tetracycline responsive control element and can be repressed by administration of tetracycline or an analogue, doxycycline [21–23].

We modified these transgenic strains so that their B-cell repertoire was dominated by a mature BCR for the antigen hen egg lysozyme (HEL), by breeding in a transgene for that receptor (BCR^HEL^), whose expression was targeted to the B cell lineage [24]. We could then provide an antigenic stimulus by breeding in a transgene that produced a soluble version of the normally membrane-bound antigen (sHEL) [25]. This set of mouse models allowed us to examine whether either autogenous or antigen-induced signaling from the BCR could cooperate with overexpressed *MYC* in the initiation and maintenance of BCLs.

Our results suggest that both forms of signaling from the BCR can cooperate with overexpressed *MYC* in tumorigenesis. The tumors that arose in mice expressing both BCR^HEL^ and sHEL differed from those found in Eμ-*MYC* mice, and bore a striking resemblance to BL. Reconstruction experiments demonstrated that both the survival and proliferation of these tumors was dependent upon the cognate autoantigen for the BCR. We also found that BCR^HEL^ itself could cooperate with *MYC* in tumorigenesis. The resulting tumors differed from both those in the Eμ-*MYC* mice and those in Eμ-*MYC*/BCR^HEL^/sHEL mice, and resembled a subset of chronic B-cell lymphocytic leukemia (B-CLL). We attribute the phenotype of the Eμ-*MYC*/BCR^HEL^ tumors to autogenous signaling from the BCR [4, 26]. The requirement for continuous BCR signals in the maintenance of either the murine BL-like tumors or the B-CLL–like tumors was authenticated by genetic and pharmacological disruption of signaling from the BCR.

Our findings provide experimental support for previous suggestions that both autogenous and antigen-stimulated signaling from the BCR can contribute to lymphomagenesis; provide a direct demonstration that autoantigenic stimulation can contribute to lymphomagenesis; and sustain the view that the constitutive BCR gives rise to autogenous signals that differ from those elicited by antigen. This is also the first report, to our knowledge, in which the potential contributions of constitutive and antigen-stimulated BCR to lymphomagenesis are compared. The models that are dependent upon autoantigenic stimulation bear a close resemblance to human large BCLs, and to BL, in particular. The results also raise the possibility that interruption of signaling from the BCR may have therapeutic value in the treatment of BCLs that express the receptor. The animal models described here should be useful in exploring further the pathogenesis of lymphomas and in preclinical testing of new therapeutics for lymphomas.

## Results

### Introduction of Antigen Specificity into B Cells Expressing a Transgene for *MYC*

To test the role of BCR signaling in lymphomagenesis, we generated mice containing B cells that both overexpressed *MYC* and had a known antigenic specificity at a high frequency. To that end, we bred a transgene for BCR^HEL^ into Eμ-*MYC* mice, creating a strain designated Eμ-*MYC*/BCR^HEL^. Expression of the Eμ-*MYC* and BCR^HEL^ transgenes was targeted to the B cell lineage [20,25]. Mice that express the Eμ-*MYC* transgene alone appear developmentally normal at first [27], but later accumulate a large number of polyclonal Pre/Pro-B cells (B220+, CD43+, IgM–, IgD–) in their bone marrow, and eventually also in their peripheral lymphoid organs, from which a monoclonal Pre/Pro BCL arises [20]. In contrast, the bone marrow and lymph nodes of Eμ-*MYC*/BCR^HEL^ mice contained normal numbers of mature B cells, which expressed BCR^HEL^ on their surface (unpublished data). Thus, the developmental arrest normally observed in Eμ-*MYC* mice was apparently corrected by the introduction of an antigen receptor transgene, in accord with previous results [28]. The Eμ-*MYC*/BCR^HEL^ mice provided a means to test for cooperation between signaling from the BCR and overexpression of *MYC* in the genesis of lymphoid tumors.

### Expression of BCR^HEL^ in the B Cell Lineage Altered Lymphomagenesis by *MYC*

**Figure 1:**
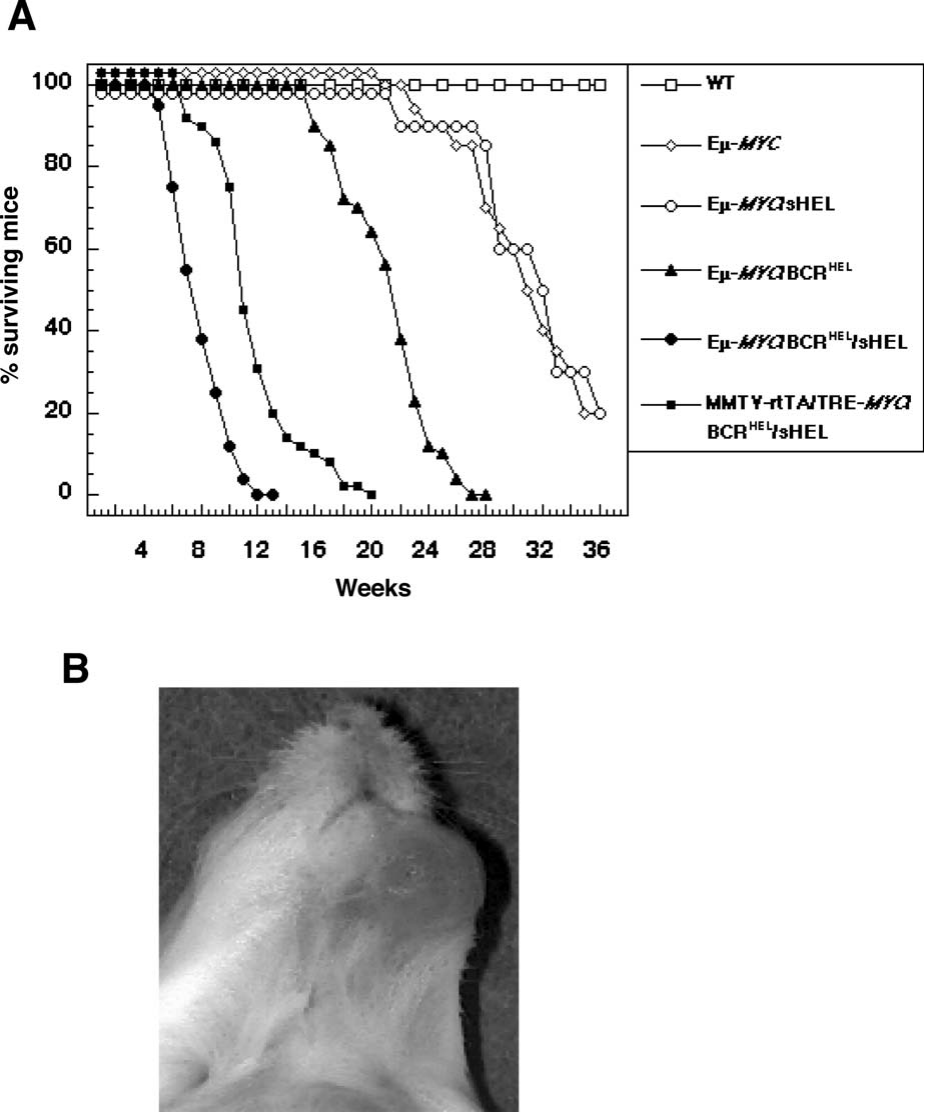
A Clonal B cell Antigen Receptor Cooperates with *MYC* in the Development of BCLs (A) Survival. Strains of mice in groups of 50 were observed over a period of 36 wk. Deceased mice were examined by necropsy. Death was uniformly attributable to lymphoid tumors. The difference among the mortality curves for the Eμ-*MYC*/BCR^HEL^/sHEL mice and that of MMTV-rtTA/TRE-*MYC*/BCR^HEL^/sHEL transgenic mice to each other had a significance value *p* = 0.05. The difference between the mortality curves for those two sets of mice and the other mice represented in the graph was *p* = 0.005. In addition, the statistical significance of the difference between the Eμ-*MYC*/BCR^HEL^ transgenic mice and any other groups of mice presented in the graph is *p* < 0.01. (B) Jaw tumor in 16-wk-old MMTV-rtTA/TRE-M*YC*/BCR^HEL^/sHEL mouse.

**Figure 2:**
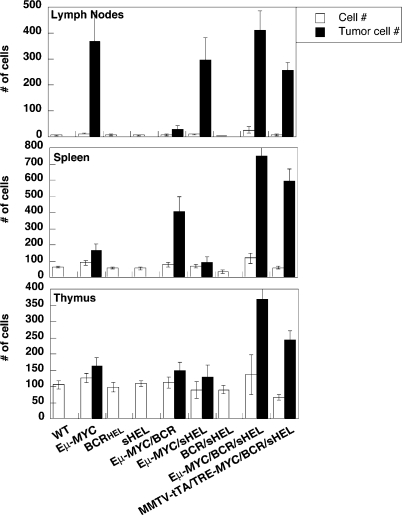
Lymphomagenesis in Transgenic Mice Single-cell suspensions were generated from lymph nodes (six nodes for each mouse – a pair of inguinal, axillary and brachial lymph nodes), spleens, thymii and jaw-tumors. The bar graphs represent the total number of cells (x10^−6^) obtained for the indicated organs. Counts represent the mean derived from 10 independent mice ± the standard deviation for those values. Healthy animals were euthanized at 21 d of age, Eμ-*MYC* mice at 200–240 d, Eμ-*MYC*/BCR^HEL^ mice at 112–130 d, Eμ-*MYC*/BCR^HEL^/sHEL mice at 26–30 d, and MMTV-rtTA/TRE-*MYC*/sHEL/BCR^HEL^ mice at 71–86 d. All tumors contained homogeneous populations of cells with distinctive surface phenotypes: B220+/IgM- cells for Eμ-*MYC* tumors, B220+/IgM^a^+ cells for both Eμ-*MYC*/BCR^HEL^ and Eμ-*MYC*/BCR^HEL^/sHEL tumors, and B220+/BCR^HEL^ cells for MMTV-rtTA/TRE-*MYC*/BCR^HEL^/sHEL tumors. Open bars represent normal mice. Filled bars represent tumor-bearing mice.

The Eμ-*MYC*/BCR^HEL^ mice developed fatal lymphomas more rapidly than did Eμ-*MYC* mice (Figure 1A), and the anatomical distribution of the tumor was different (Figure 2). These observations are based on the detailed analysis of tumor that arose in 80 individual mice, maintained in three different animal facilities in two institutions. We consistently observed this phenotype in the tumors, in spite of previous reports of some low frequency of mature B cell malignancies [29,30]. The emergence of tumors was followed in three ways: by anatomical inspection, by counting the total number of cells in organs (Figure 2), and by flow cytometric analysis to enumerate B cells carrying BCR^HEL^ (Figure S1).

**Figure 3:**
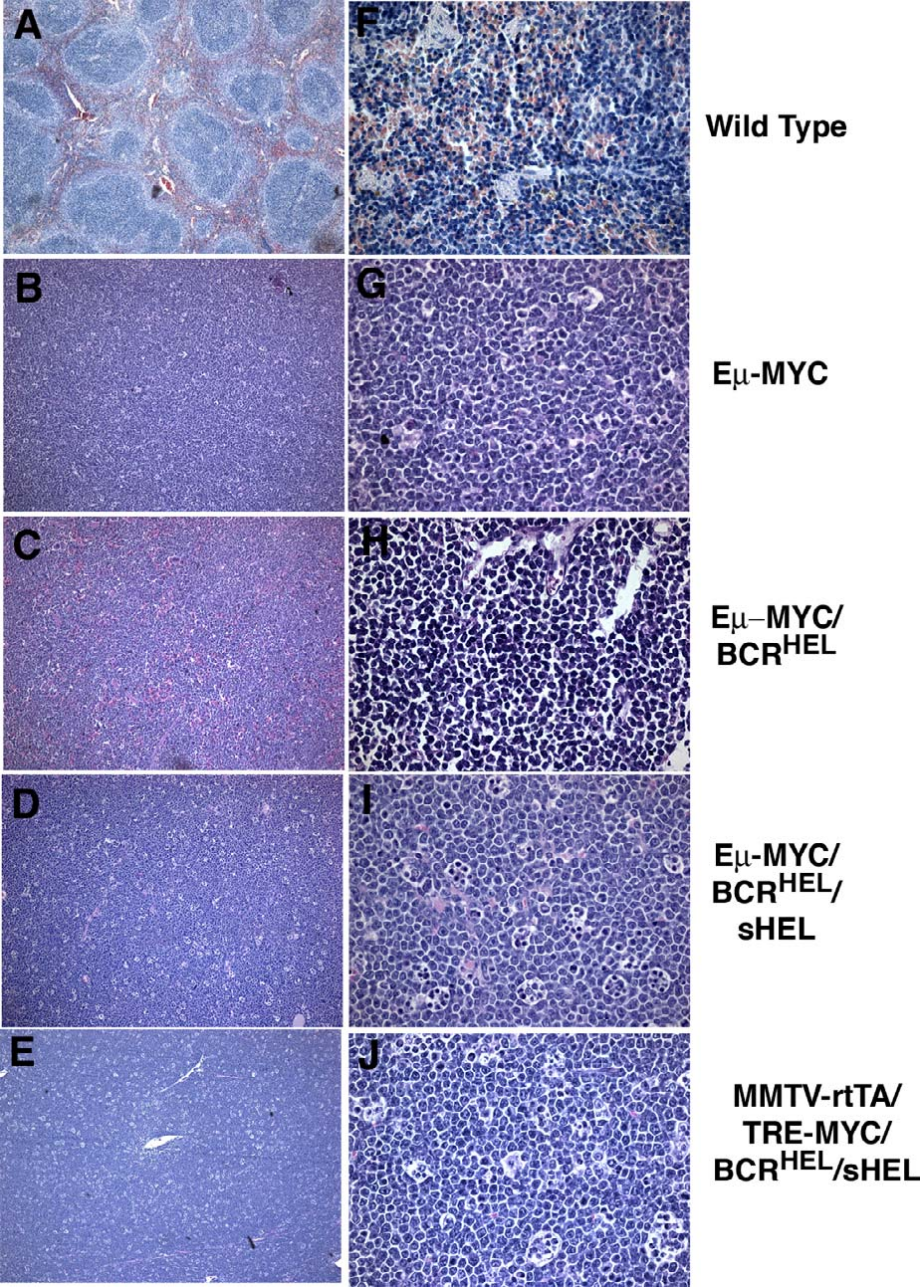
Histological Analysis of Tumors Tissues were sectioned, stained with hematoxylin and eosin, and microscopic images obtained as described in Methods. Magnification was 10X for (A–E), 100X for (F–J). (A and F) Spleen from a normal wild-type mouse. (B and G) Lymph node tumor from an Eμ-*MYC* mouse. (C and H) Spleen tumor from an Eμ-*MYC*/BCR^HEL^ mouse. (D and I) Spleen tumor from an Eμ-*MYC*/BCR^HEL^/sHEL mouse. (E and J) Jaw tumor from an MMTV-rtTA/TRE-*MYC*/BCR^HEL^/sHEL mouse.

Evidence of tumor in Eμ-*MYC*/BCR^HEL^ mice appeared first in the spleen at about 18 wk of age, then in lymph nodes and the bone marrow. Histological examination of the tumors revealed a diffuse and homogeneous population of small lymphocytes (Figure 3C and 3H). Analysis with a panel of cell-surface markers identified the tumor cells as mature but naïve B cells, reminiscent of those found in a subset of human B-CLL (Table 1 and Figure S1), whereas the Eμ-*MYC* tumors were composed of Pre/Pro B-cells (Table 1, in accord with [20]). In addition, the tumors in Eμ-*MYC*/BCR^HEL^ mice appear to be mature naïve B cells that are CD5–. In human B-CLL, the two main subsets are CD5+ and CD5–. Tumors composed of mature, naïve B cells also arose in MMTV-rtTA/TRE-*MYC*/BCR^HEL^ mice that had not been exposed to doxycycline (unpublished data). Those mice overexpress *MYC* from a different control element, but developed the same sort of tumors as observed with the Eμ-*MYC*/BCR^HEL^ transgenes (Table 1 and Table S1). We conclude that a constitutive BCR can cooperate with *MYC* in the genesis of BCLs and can elicit a distinctive phenotype in the tumor cells. We attribute the findings to a previously described form of autogenous signaling from the BCR [4,26] (See Discussion).

**Table 1:**
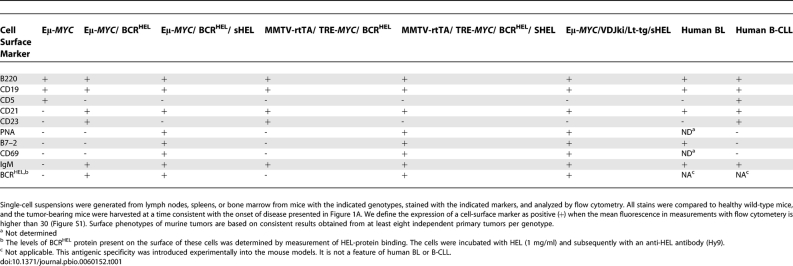
Cell-Surface Markers of BCLs

### Antigenic Stimulation Altered Lymphomagenesis by *MYC*

To explore how antigen stimulation of BCR^HEL^ might affect tumorigenesis by *MYC*, we bred a ubiquitously expressed transgene for sHEL into the Eμ-*MYC*/BCR^HEL^ background. The resulting strain (Eμ-*MYC*/BCR^HEL^/sHEL) developed tumors even more rapidly than did Eμ-*MYC*/BCR^HEL^ mice (Figure 1A).

Overgrowth of B cells could be detected in the bone marrow, lymph nodes, spleen, and thymus (Figure 2). B cells also infiltrated the liver, lungs, and central nervous system. Compression and invasion of the spinal cord caused paralysis of the hind and fore limbs. Histological examination revealed a homogeneous population of large lymphocytes in the spleen, lymph nodes, thymus, and bone marrow. The sheets of cells had a “starry sky” appearance (Figure 3D and 3I) that is common among large BCLs and is a prominent feature of BL [31]. This designation results from the presence of sheets of monomorphic cells interspersed with macrophages that have engulfed apoptotic cells. When examined for surface markers, the tumor cells closely resembled mature, activated B lymphocytes that have experienced the germinal center (GC). Further evidence for the GC origins of these cells will be presented below. As expected, the tumor cells were specific for HEL, as evidenced by their ability to bind the antigen (Table 1).

We also bred the BCR^HEL^ and sHEL transgenes into a second strain of mice that expresses *MYC* in the B cell lineage (MMTV-rtTA/TRE-*MYC*)(see Introduction and Materials and Methods). The final composite strain was designated MMTV-rtTA/TRE-*MYC*/BCR^HEL^/sHEL. We originally created these mice for other purposes, but the manner in which they developed tumors proved noteworthy for the present context. The mice died somewhat later than the Eμ-*MYC*/BCR^HEL^/sHEL mice, but earlier than the other strains analyzed in the present study (Figure 1A). In a striking departure from our previous experience, however, tumors appeared first in the jaw, in a randomly unilateral manner (Figure 1B). The mice eventually developed a more generalized disease, with tumor cells appearing in multiple lymphoid organs and infiltrating nonlymphoid tissues as well (Figure 2 and unpublished data). We observed this phenotype in 53 of 60 mice that were analyzed. The remaining seven mice in that cohort of 60 animals was only found to be sick at a very advanced stage of the disease, so we can not formally state that they initially presented with a randomly unilateral tumor in the jaw. The histological appearance of the tumors was similar to that of the Eμ-*MYC*/BCR^HEL^/sHEL tumors, including a starry sky appearance (Figure 3E and 3J). The surface phenotype of MMTV-rtTA/TRE-*MYC*/BCR^HEL^/sHEL tumor cells also resembled that of Eμ-*MYC*/BCR^HEL^/sHEL tumors (Table 1 and Figure S1). The jaw tumors were covered with a thin layer of calcified material (unpublished data), a feature not associated with tumors at other sites or in the other strains of mice. The endemic form of BL initially presents in a randomly unilateral manner in the jaw (see Discussion).

In summary, the constitutive and antigen-stimulated forms of BCR^HEL^ altered tumorigenesis by *MYC* in distinctive manners. The distinctions involved diverse features of the tumors, including rate of appearance, anatomical presentation and progression, histopathology, and cell-surface phenotype (Table S1). The tumors that arose under the influence of a constitutive BCR resembled human B-CLL, whereas those that developed in the presence of antigen stimulation resembled BL in multiple ways and were similar in two strains of mice with different *MYC* transgenes. Importantly, our work involves the overexpression of *MYC* in the context of autoreactive B cells. The overexpression of *MYC* is the characteristic genetic lesion in BL, such that these mouse models are the most germane to the mechanisms that give rise to BL.

### Tumors Driven by Antigen Receptor Signals Are Oligoclonal

To determine the clonality of the various tumors, we used PCR to analyze the V_H_ to DJ_H_ rearrangements of the endogenous *IgH* genes. The results are summarized in Table 2 and documented in Figure S2. As expected, the data from spleen cells of normal mice revealed evidence of germ line IgH genes, as well as innumerable rearranged configurations. Similar results were obtained with spleen cells obtained from an MRL*^lpr/lpr^* mouse with a characteristic lymphoproliferative disease that involves innumerable B cell clones [32]. In contrast, tumors derived from the various strains of mice with *MYC* transgenes were composed of relatively few clones of B cells and displayed no evidence of germ line configurations. The Eμ-*MYC* tumors were largely monoclonal (unpublished data), as reported previously [20]. The Eμ-*MYC*/BCR^HEL^ and Eμ-*MYC*/BCR^HEL^/sHEL tumors contained multiple clones, with the latter slightly more complex than the former (20–40 discrete clones for the Eμ-*MYC*/BCR^HEL^ tumors, and 10–15 clones for the Eμ-*MYC*/BCR^HEL^/sHEL tumors). Tumors in both the jaw and other sites of the MMTV-rtTA/TRE-*MYC*/BCR^HEL^/sHEL mice were also multiclonal, and in any given animal, the patterns of Ig rearrangement were similar in tumors from all sites, suggesting that the same group of neoplastic clones gave rise to all of the tumors in the animal (unpublished data). The same conservation of clonal patterns among different organ sites was observed for tumors derived from the other genotypes (Eμ-*MYC*, Eμ-*MYC*/BCR^HEL^, and Eμ-*MYC*/BCR^HEL^/sHEL).

**Table 2:**
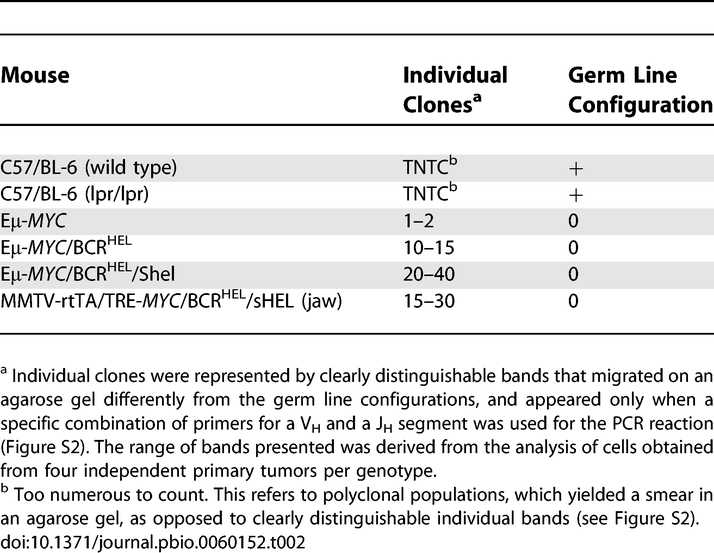
The Clonality of Tumors

We conclude that tumors of Eμ-*MYC*/BCR^HEL^, Eμ-*MYC*/BCR^HEL^/sHEL and MMTV-rtTA/TRE-*MYC*/BCR^HEL^/sHEL mice arose from a limited number of B cell clones. The multiclonality of the tumors sets them apart from their ostensible human counterparts, which are typically monoclonal [31]. We attribute this clonal dominance to selection of clones in which even further tumorigenic events have occurred (see Discussion).

### Antigen Dependence of Tumors

We wanted to explore the specific contribution of antigen-dependent signaling to lymphoid transformation in the Eμ-*MYC*/BCR^HEL^/sHEL tumors. As a first approach, we asked whether the exogenous antigen (HEL) could alter phenotypically normal MMTV-tTA/TRE-*MYC*/BCR^HEL^ cells to resemble the tumor cells of

**Figure 4:**
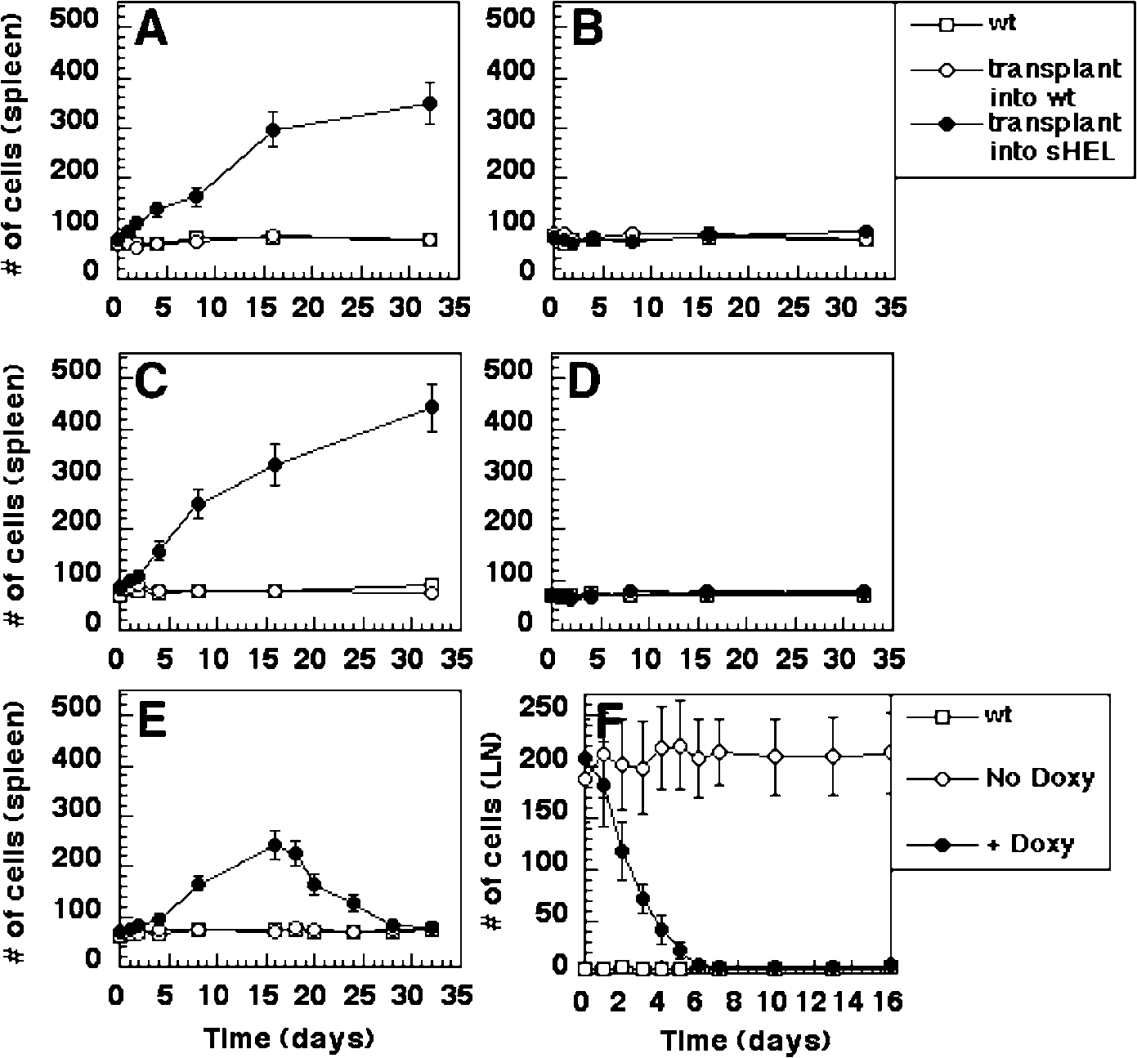
Establishment and Maintenance of Murine BL by Antigenic Stimulus and *MYC* Overexpression (A and B) Primary transplants. Spleen and lymph node cells were harvested from an MMTV-rtTA/TRE-*MYC*/BCR^HEL^ mouse at 4 wk of age. Cells from spleen and lymph nodes were pooled at a 1:1 ratio, and 10^6^ cells were introduced into either syngeneic wild-type mice (empty circles) or sHEL transgenic mice (filled circles) by intravenous injection. Cohorts of mice were either kept on regular food (A), or on doxycycline-containing food (B). Tissues were collected at indicated time points from spleens and analyzed for total number of cells. Samples taken from wild-type mice were analyzed at the same times (empty squares). (C and D) Secondary transplants. Cells were collected from tumors of spleens and lymph nodes represented in (A), 16 d after their initiation by transplantation. Cells from spleen and lymph nodes were pooled at a 1:1 ratio, and 10^5^ cells were introduced into either wild-type recipients (empty circles) or sHEL transgenic mice (filled circles) by intravenous injection. The empty squares represent wild-type, unmanipulated mice that were analyzed in parallel with the experimental groups. Cohorts of mice were either kept on regular food (C), or on doxycycline-containing food (D). Cells were collected from spleens at the indicated times after the transplantation and analyzed as in (A and B). (E and F) BCLs regress after *MYC* overexpression is extinguished. (E) A cohort of mice similar to those described in (A) was allowed to develop externally visible lymphadenopathy. 16 d later, those mice were switched to a doxycycline-containing diet. The empty circles represent wild-type recipient mice that received transplants of MMTV-rtTA/TRE-*MYC*/BCR^HEL^ cells, the filled circles represent sHEL transgenic mice that received transplants of those cells, the empty squares represent wild-type, unmanipulated mice that were analyzed in these experiments in parallel with the experimental mice. Cells were collected from spleens at the indicated times after the transplantation and analyzed as in (A and B). (F) MMTV-rtTA/TRE-*MYC*/BCR^HEL^/sHEL mice were allowed to develop tumors spontaneously, as a result of transgene function. Approximately 40 d later, mice with externally apparent lymphadenopathy were given doxycycline containing food (day 0 in figure). Cells were collected from lymph nodes at the indicated times after exposure of the mice to doxycycline and analyzed as in (A and B). The empty circles represent MMTV-rtTA/TRE-*MYC*/BCR^HEL^ mice that were never exposed to docycycline, the filled circles represent MMTV-rtTA/TRE-*MYC*/BCR^HEL^ mice that were given doxycycline-containing food after they developed externally apparent lymphadenopathy, the empty squares represent wild-type, unmanipulated mice that were analyzed in parallel with the experimental mice.

Eμ-*MYC*/BCR^HEL^/sHEL and MMTV-rtTA/TRE-*MYC*/BCR^HEL^/sHEL mice. Cells were taken from 4–6-wk-old MMTV-rtTA/TRE-*MYC*/BCR^HEL^ mice that had been fed doxycycline-containing food since birth in order to suppress expression of the *MYC* transgene (Figure S3). The donor animals appeared clinically normal, and had normal numbers of cells in their lymph nodes and spleen (unpublished data). The donor cells were tracked throughout the experiment by flow cytometric analysis for a B cell marker (B220) and the BCR^HEL^. Donor cells were transferred to either C57/BL6 mice or age and sex matched mice carrying only the transgene for sHEL. The recipient mice were not treated with doxycycline to allow activation of the *MYC* transgene in the donor cells. In the absence of sHEL, the number of donor cells detectable in recipient mice did not change (Figure 4A) and the mice remained healthy. In contrast, the number of donor cells in sHEL recipients rose continuously over time (Figure 4A) until the mice developed overt tumors, 8–12 d after receiving the transplanted cells, and eventually died within 28–30 d.

In order to test the durability of the requirement for antigen, we harvested tumor cells that had arisen after the initial transplantation of MMTV-rtTA/TRE-*MYC*/BCR^HEL^ cells into sHEL mice and introduced these into either wild-type or a second set of sHEL mice that were not treated with doxycycline. Donor cells again amplified rapidly in the sHEL mice, but not in wild-type recipients (Figure 4C). Tumors resulted in death of the recipient mice within 12–16 d, a latency appreciably shorter than that observed after the preceding transplantation (28–30 d).

The flow cytometric profile of the donor cells changed after their encounter with antigen. The donor cells expressed B220, BCR^HEL^, CD19, CD21, and CD23, but did not express CD69 or B7–2 on their surface. The cells that resulted from the expansion following antigenic stimulation in vivo showed a loss of CD23 expression and high levels of CD69 and B7–2 expression (unpublished data). The same phenotype was observed with the cells that had expanded following the second transplantation into sHEL recipient mice. This phenotype is typical of antigen-activated B cells. In addition, it is similar to what we initially observed in the cells from tumors that arose in the Eμ-*MYC*/BCR^HEL^/sHEL mice and, thus, also resembles the phenotype of BL (Table 1). We conclude that the appearance and expansion of HEL-specific, BL-like tumor cells in the recipient mice were dependent on stimulation by the cognate antigen.

In contrast to the preceding findings, tumor cells obtained from Eμ-*MYC*/BCR^HEL^/sHEL mice or MMTV-rtTA/TRE-*MYC*/BCR^HEL^/sHEL mice would grow into lethal tumors when transplanted into recipient mice in the absence of HEL antigen (Figure 4A, 4C, and 4F). We postulated that this seeming independence of antigen was due to the intrinsic production of antigen by the tumor cells themselves, as would be expected from the genotype of the cells. That proved to be the case. We were able to detect HEL transcripts and HEL protein in cells obtained from either a primary Eμ-*MYC*/BCR^HEL^/sHEL tumor or an MMTV-TRE-*MYC*/BCR^HEL^/sHEL tumor, but not in cells obtained from either Eμ-*MYC* or Eμ-*MYC*/BCR^HEL^ tumors (unpublished data). Similarly, we found HEL protein in tumors that arose following transplantation of cells from a primary Eμ-*MYC*/BCR^HEL^/sHEL tumor. We conclude that the murine BL cells are in fact antigen-dependent, but can be sustained by either autocrine or paracrine stimulus.

### Mature B Cell Tumors Require Continuous Signaling from the BCR

**Figure 5:**
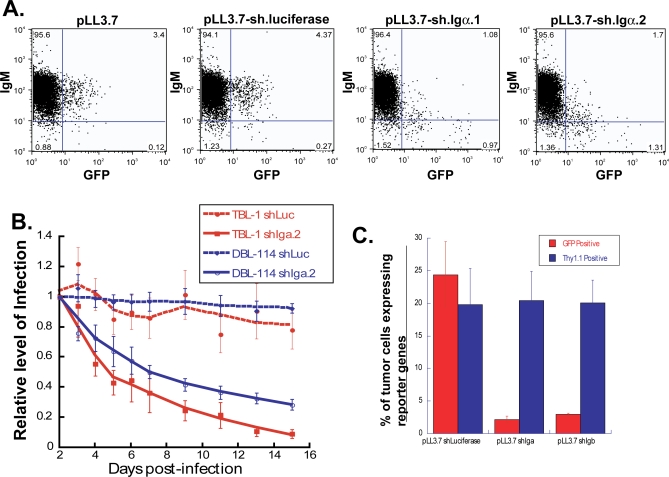
The Maintenance of Tumors Derived from Eμ-MYC/BCR^HEL^ and Eμ-*MYC*/BCR^HEL^/sHEL Mice Depends on the Continued Expression of Igα or Igβ (A) Cell lines were generated from either Eμ-*MYC*/BCR^HEL^ tumors and designated as DBL114, or from Eμ-*MYC*/BCR^HEL^/sHEL tumors and designated TBL-1. These cell lines uniformly express B220 and IgM on their surface. To determine whether the shRNA sequences targeting Igα were able to knock down their target protein, we measured the levels of IgM expressed on the surface of DBL-114 cells that were transduced with lentiviral constructs that encode a reporter gene (*GFP*). The expression of IgM on the surface is co-modulated with Igα expression, hence the loss of Igα should reduce the levels of surface IgM. The panels represent the flow cytometric profile of the DBL-114 cells that had been transduced with the parental virus (pLL3.7), or a variant that encodes an shRNA specific for firefly luciferase, as a negative control, or two variants of pLL3.7 that encode different shRNAs specific for Igα. The GFP-positive cells in the panels that contained shRNAs specific for Igα showed a loss of surface IgM expression. This is not the case for the GFP-negative fraction of the same cell populations. Similar results were obtained in TBL-1 cells (unpublished data). (B) The shRNA-mediated knock-down of Igα or Igβ in cells obtained from either Eμ-*MYC*/BCR^HEL^ or Eμ-*MYC*/BCR^HEL^/sHEL tumors confers a competitive disadvantage on those cells in vitro, compared to their nontransduced counterparts. Single-cell suspensions were generated from the respective tumors, and used for lentiviral transductions. The cells were maintained in cultured and assayed for GFP expression, by flow cytometry every 24 h. The data for the GFP+ fraction in the population of cells harboring a lentivirus encoding and shRNA was divided by the fraction of GFP+ cells in the population of cells that was transduced with the parental vector, in order to standardize the values and examine the rates of change from the starting level of GFP+ cells, as previously reported [104]. The cells that were transduced with lentiviruses encoding shRNAs specific for either Igα or Igβ exhibited a significant competitive disadvantage when compared to the cells harboring lentiviruses encoding shRNAs specific for firefly luciferase. All wells were set up in triplicates. The graphs represent data from one experiment, representative of eight independent experiments. (C) In vivo validation of the effects of Igα-specific shRNAs on the maintenance of Eμ-*MYC*/BCR^HEL^ tumors. Cells were obtained from Eμ-MYC/BCR^HEL^ tumors, and transduced in vitro with pLL3.77-sh.luciferase (uses thy1.1 as a reporter gene) or pLL3.7-sh.Igα.1 (uses GFP as a reporter gene). The different cell populations were then mixed in order to generate mixtures of cells that contained an approximately equal fraction of cells that harbored the control lentivirus (pLL3.77.sh.luciferase) and the experimental lentivirus (pLL3.7.sh.Igα). The mixtures of cells were transplanted into cohorts of Rag-1^–/–^ mice. The mice were observed daily until they exhibited externally evident signs of lymphoma, and the organs were harvested. The graphs represent the fraction of cells in the tumorous lymph nodes that retained expression of either thy1.1 (for the control lentivirus), or GFP (for the Igα-specific lentivirus. These results confirm the requirement for Igα expression in the maintenance of the murine BCLs.

To further test the role of antigen stimulation in the genesis of the B-CLL– and BL-like tumors, we disrupted the molecular machinery that generates and transmits signals from the BCR. By transduction of suitable interfering RNAs into established tumor cells, we were able to suppress the expression of the Igα and Igβ signaling chains of the BCR. We have previously shown that these shRNA sequences targeting Igα and Igβ lead to substantial reduction in the levels of Igα or Igβ protein in K46μ B cells, respectively [33]. We have observed that shRNAs directed at either Igα or Igβ individually caused a decrease in the levels of surface IgM in transduced B cells. Importantly, the expression of Igα and Igβ on the cell surface modulates the surface expression of IgH and IgL, the antigen-specific components of the BCR. The vector used for transduction was a lentivirus that has been previously described [34]. The viruses tested in vitro and in vivo were the parental vector encoding either GFP (pLL3.7) or Thy1.1 (pLL3.77) as a reporter gene, and variants encoding both a reporter gene and shRNAs for either Igα, Igβ, or firefly luciferase, the last as a negative control. Transduction was performed with tumor cells isolated from either Eμ-*MYC*/BCR^HEL^ mice or Eμ-*MYC*/BCR^HEL^/sHEL mice. The rates of transduction varied between 5%–50% of the tumor cells (Figure 5A). The cells were then maintained in culture and evaluated on a daily basis for reporter gene expression.

We observed that for both Eμ-*MYC*/BCR^HEL^ and Eμ-*MYC*/BCR^HEL^/sHEL tumors, the percentage of cells transduced with either the parental lentivirus, or the variant that encoded shRNA specific to firefly luciferase, did not change appreciably during a 14 day period (Figure 5B). In contrast, each of the cultures that was transduced with a lentiviral variant that encoded shRNAs specific to either Igα or Igβ displayed a significant decrease in the frequency of the cells expressing the reporter gene (Figure 5B). The same pattern was observed with two independent shRNAs for each of Igα and Igβ. This in vitro competition assay suggested that loss of signaling from the BCR placed the tumor cells at a significant disadvantage when compared to their counterparts that had retained normal signaling from the BCR. In addition, these results demonstrate that the signaling components of the BCR are required for tumors that depend on autogenous signaling by the BCR-derived signals as well as for those that rely on the cognate-antigen–induced BCR signals.

To verify these observations in vivo, we isolated tumor cells from Eμ-*MYC*/BCR^HEL^ mice, and transduced them as described above. We then transplanted the transduced tumor cells into Rag-1^–/–^ mice, to evaluate tumor fitness in the absence of any T cell responses to the reporter genes. In this instance, we mixed tumor cells that had been transduced with pLL3.77 (providing thy1.1 as a reporter) encoding an shRNA to firefly luciferase with tumor cells transduced with pLL3.7 (providing GFP as a reporter) encoding shRNAs specific to either Igα or Igβ. This would provide internal controls for each of the mice we transplanted in the cohort. We euthanized the mice 21 days after transplantation, when they developed external signs associated with lymphoma (scruffy fur, hunched posture, lymphadenopathy, dehydration, labored breathing, and an ascending hind limb paralysis). The lymph nodes and spleens were collected and used to generate single-cell suspensions. The cells were then stained and analyzed by flow cytometry.

Tumor cells transduced with either pLL3.7-sh.Igα or pLL3.7-shIgβ failed to expand in vivo (Figure 5C), mirroring the results obtained in vitro. In contrast, the nontransduced cells, or the cells transduced with pLL3.77-sh.Luc, expanded in vivo and gave rise to the resulting malignancies. In addition, the genetic disruption of Syk, a key membrane-proximal element of the BCR signal, also conferred a significant competitive disadvantage to established BCL cells in a manner analogous to what we present here with Igα or Igβ (RMY and YR, unpublished results). These results show that for both tumor types, the acute loss of the signaling components of the BCR complex resulted in a severe competitive disadvantage in vivo, suggesting that these two types of tumors are dependent upon continuous signaling from the BCR.

### The Response of Tumors to Immunosuppressants

The apparent contribution of BCR signaling to the development of murine lymphomas prompted us to explore the effect of immunosuppressants on the various mouse models. We used cyclosporin A, FK506, and rapamycin to treat well advanced tumors that had been initiated by transplantation. We compared the effects of these agents to that of cyclophosphamide, an agent commonly used to treat human BL [35].

We transplanted 10^6^ cells obtained from tumor-bearing spleen or lymph nodes into cohorts of 4–10 mice. The recipient mice were held for observation until they developed externally obvious lymphadenopathy (approximately 100 d for the Eμ-*MYC* tumors, 58 d for the Eμ-*MYC*/BCR^HEL^ tumors, 21 d for the Eμ-*MYC*/BCR^HEL^/sHEL tumors, and 14 d for the MMTV-rtTA/TRE-*MYC*/BCR^HEL^/sHEL tumors). The tumor bearing and control wild-type mice were then treated daily for 7 d with intravenous injections of the various drugs. Mice were either euthanized 24 h after the last injection of drug, or held indefinitely to ascertain duration of survival. The analysis of tumor burden was performed with cells obtained from lymph nodes and spleens.

**Figure 6:**
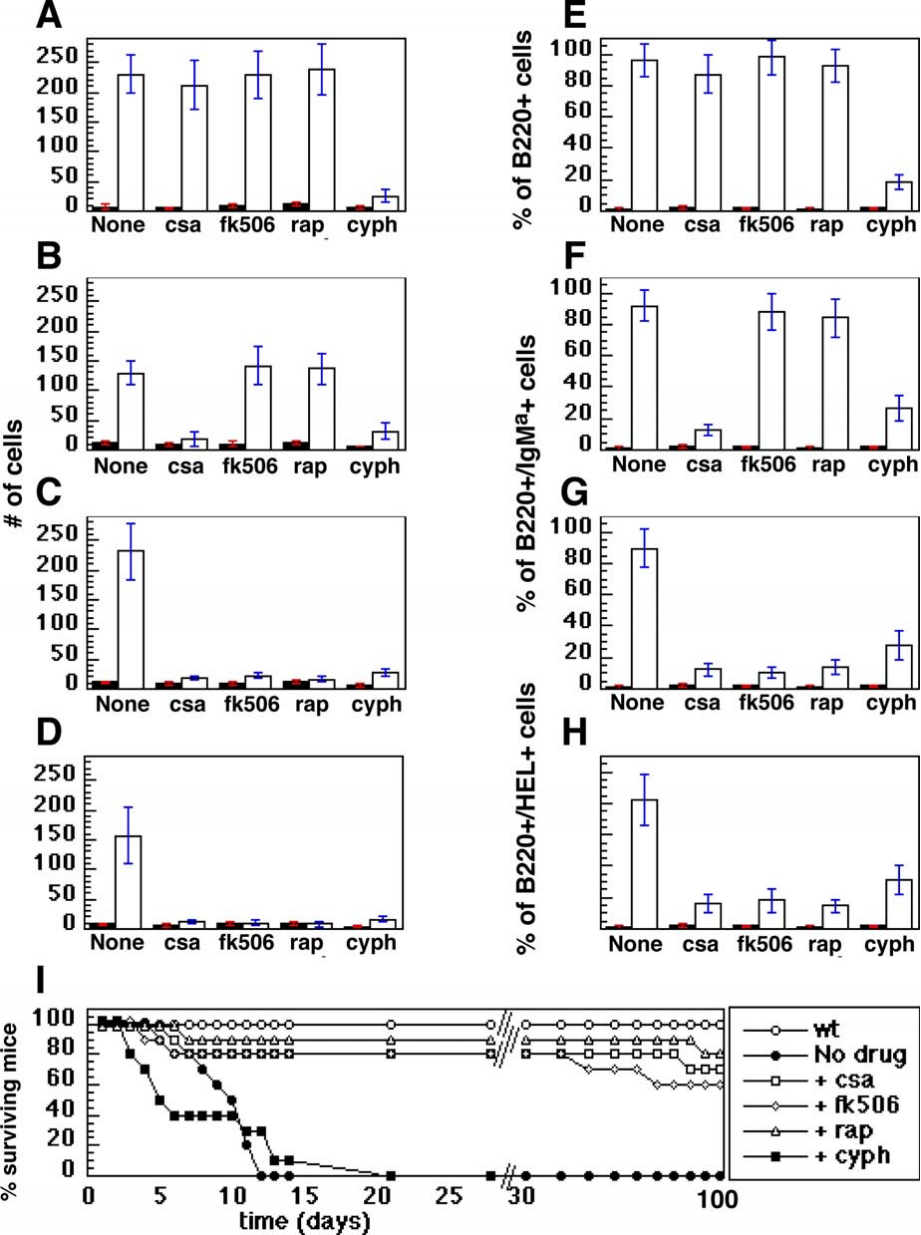
Suppression of Tumor Growth by Pharmacological Agents Tumor cells were harvested from lymph nodes and spleens and transplanted as described in Methods. The recipient mice were held until tumors became clinically apparent. Tumor recipient (open bars) and wild type (filled bars) mice then received daily injections of the indicated drugs for 7 d of either cyclosporine A (csa), FK506, rapamycin (rap), or cyclophosphamide (cyph). For (A–H), the mice were euthanized 24 h after the last injection of drug, and lymph nodes were harvested for analysis of either total number of cells (A–D) (expressed in single units representing 10^6^ cells each) or surface markers of donor cells (E–H). For (I), the mice were observed over a span of 100 da and deaths recorded, as shown. (A and E) Eμ-*MYC* tumors. (B and F) Eμ-*MYC*/BCR^HEL^ tumors. (C and G) Eμ-*MYC*/BCR^HEL^/sHEL tumors. (D and H) MMTV-rtTA/TRE-*MYC*/BCR^HEL^/sHEL tumors. (I) Survival of animals bearing Eμ-*MYC*/BCR^HEL^/sHEL tumors. The statistical significance of the differences observed in the kinetics of mortality between the tumor-bearing mice that were either untreated, or treated transiently with cyclophosphamide is 0.01. The statistical significance of the difference in the mortality curves observed between those two groups and the tumor-bearing mice treated with either of the three immunosuppressant drugs is *p* < 0.001.

The Eμ-*MYC* tumors did not respond to any of the immunosuppressive drugs we tested (Figure 6A and 6E). Disease progressed at the same rate in treated and untreated mice. Histological examination of the affected organs also revealed no evidence of therapeutic response (unpublished data). In contrast, the transplanted Eμ-*MYC* tumors showed a strong response to cyclophosphamide, as previously described [36]. Treatment with cyclophosphamide elicited tumor regression in all animals, but also caused a more general cytotoxicity, manifested as a reduction in T cells, myeloid cells, and nontransgenic B cells (unpublished data). Similar toxicity from cyclophosphamide was also observed in wild-type mice.

The tumors derived from the Eμ-*MYC*/BCR^HEL^ mice responded to cyclophosphamide and cyclosporin, but not to either FK506 or rapamycin (Figure 6B and 6F). In contrast, the tumors from Eμ-*MYC*/BCR^HEL^/sHEL mice, as well as tumors from the jaws of MMTV-rtTA/TRE-*MYC*/BCR^HEL^/sHEL mice, responded to both cyclophosphamide and all three of the immunosuppressants tested (Figure 6C, 6D, 6G, 6H). The different sensitivities of the tumors derived from Eμ-*MYC*/BCR^HEL^ mice and those derived from either Eμ-*MYC*/BCR^HEL^/sHEL or MMTV-rtTA/TRE-*MYC*/BCR^HEL^/sHEL mice suggests that the constitutive and cognate-antigen–derived BCR signals may be qualitatively distinct.

Remissions of Eμ-*MYC*/BCR^HEL^/sHEL tumors persisted for at least 5 mo, following a 7-d course of treatment with immunosuppressants (Figure 6I and unpublished data). In contrast, the animals treated with cyclophosphamide entered a brief remission, but still died more rapidly than did untreated, tumor-bearing mice (Figure 6I and unpublished data), apparently consequent to the toxicity described above.

In summary, the response of the various model tumors varied consistently with the genotypes of the mice. B cell tumors that arose from the combined effects of *MYC* and antigen stimulus responded uniformly to three distinctive immunosuppressants, whereas only one of the three agents was effective against tumors elicited by *MYC* and a constitutive BCR (For data demonstrating the post-GC nature of antigen-stimulated tumors, see Figure S4). Since the Eμ-*MYC* tumors do not express BCR on their surface, they were presumably devoid of any apparent stimulus from the receptor, and were resistant to all the immunosuppressants tested. These results are in accord with two of our hypotheses: that tumorigenesis can be influenced by signals from the BCR, and that the signals generated by constitutive receptor may differ from those arising from an antigen-stimulated receptor (see Discussion).

### *MYC* Dependence of Tumors

We also explored whether the murine BL-like tumors were dependent on the continuous overexpression of *MYC*. To test this issue on established tumors, we allowed MMTV-rtTA/TRE-*MYC*/BCR^HEL^/sHEL mice to develop tumors in the absence of doxycycline. The mice were then given doxycycline-containing food in order to suppress the expression of the *MYC* transgene (Figure S3). The tumors quickly regressed (Figure 4F and unpublished data).

The establishment and maintenance of the BL-like tumors that arose following transplantation of MMTV-rtTA/TRE-*MYC*/BCR^HEL^ cells into sHEL mice was also dependent upon the overexpression of *MYC*. This conclusion was based on several findings. First, the transplantation of cells MMTV-rtTA/TRE-*MYC*/BCR^HEL^ could not give rise to tumors if the transplanted cells were prohibited from expressing their *MYC* transgene by administration of doxycycline to the recipient mice (Figure 4B). Second, tumor cells that had arisen from the transplantation of MMTV-rtTA/TRE-*MYC*/BCR^HEL^ cells could not be further passaged in the absence of overexpressed *MYC* (Figure 4D). And third, tumors that arose following transplantation of MMTV-rtTA/TRE-*MYC*/BCR^HEL^ cells would regress once expression of the *MYC* transgene was extinguished with doxycycline (Figure 4E). We conclude that the development and maintenance of the BL-like tumors requires continuous stimulation by *MYC*.

## Discussion

Previous observations have raised the possibility that expression of a BCR can contribute to the genesis of lymphomas (reviewed in [3]). We have used transgenic mice to explore this possibility. Our results demonstrated that both the constitutive and antigen-stimulated states of the BCR can cooperate with overexpression of the proto-oncogene *MYC* in the genesis of BCLs. The types of B cell tumors produced in the two instances differ appreciably, with the former resembling a subset of human B-CLL, the latter resembling human BL. This work provides a direct demonstration that signaling from the BCR can contribute to lymphomagenesis. The mouse models described here should be useful for further study of tumorigenesis in the B cell lineage, and for preclinical testing of therapeutics.

### The BCR Can Contribute to Lymphomagenesis in Both the Absence and Presence of Cognate Antigen

Previous work has shown that overexpression of *MYC* in the B cell lineage can give rise to lymphomas in mice (reviewed in [20]). We have now found that the BCR can both accelerate tumorigenesis by *MYC* and alter the nature of the resulting tumors. The acceleration is greater when the BCR is stimulated by antigen than when it is not, and the resulting tumors differ in their clinical behavior, anatomical presentation, histopathology, and surface phenotype. In addition, the two sorts of tumors respond differently to a trio of immunosuppressants, each of which can probably act on distinct signaling pathways activated by antigen receptor [37–39]. We attribute these differences among the tumors to the fact that the BCR can apparently generate at least two forms of signals—one produced by the binding of the cognate antigen, the other an “autogenous” signal generated by the ostensibly unstimulated receptor [5]. Our results sustain the view that the two forms of signals are different, suggest that this difference affects the nature of the tumors produced by *MYC*, and provide direct evidence that each of the two forms of signaling can contribute to lymphomagenesis—a notion that has heretofore been based solely on correlative data.

The biochemical nature of the constitutive signal derived from the BCR remains to be determined. Our genetic studies involving the disruption of Igα or Igβ in established tumors suggests that both of those signaling chains are required for constitutive and cognate antigen triggered signals. The results we obtained showing differential sensitivity to cyclosporine A, FK506, and rapamycin in murine BL- and B-CLL–like tumors suggest that the two signals derived from the BCR involve the activation of different transduction pathways. The tumor models we have developed may also enable us to dissect the molecular basis of constitutive BCR signaling in the context of B cell development and neoplasia.

Signaling by the antigen-stimulated BCR has been studied in great depth [40]. It is known to mediate B cell proliferation and could contribute to tumorigenesis if sustained inappropriately. We have shown here that antigen-stimulated signaling from the BCR can cooperate with the oncogene *MYC* to produce tumors that remain dependent upon antigen and that resemble BL of humans. Yan et al. recently reported that autoantigenic drive may contribute to the genesis of a tumor resembling CD5+ B-CLL in mice expressing a transgene of TCL1 [41]. These authors suggested a link to BCR signaling, based on their analysis of endogenous BCR usage. The presence of repeated patterns may be suggestive of conserved antigenic stimulation, but is not definitive. Whether a common specificity is a foreign or self-antigen is also unclear from the pattern of BCR usage. A second tcl-1 transgenic mouse presented with a tumor phenotype of large BCLs (BL, diffuse large B-cell lymphoma (DLBCL)). Those tumors were all CD5^–^, suggesting an inherent difference the two transgenic mouse strains that overexpress tcl-1 in both mature B-cell compartments [42]. A Bcl-6 transgenic mouse has also been reported to yield DLBCL-like tumors in mice that presented with a post-GC phenotype [43]. Importantly, our work involves the overexpression of *MYC* in the context of autoreactive B cells. The overexpression of *MYC* is the characteristic genetic lesion in BL, such that these mouse models are the most germane to the mechanisms that give rise to BL.

Two recent reports have also implicated autoantigenic stimulation of B cells either in the genesis of a lymphoma that occurs in mice expressing a transgene for the *TCL1* gene, and that resembles human B-CLL [41], or in the instance of DLBCL-like tumors that arose in Bcl-6 transgenic mice [43]. Subsequent analysis of the DLBCL-like tumors that develop in the Bcl-6 transgenic mice demonstrated a need for AID expression for the genesis of those post-GC B cell malignancies [44]. Our work provides a direct demonstration that autoantigenic stimulation can contribute to lymphomagenesis. The comparison of the constitutive and cognate-antigen–stimulated BCR is unique in the context of tumorigenesis models. In fact, the models we have developed represent the closest genotypic and phenotypic approximation to human BL.

In contrast, the autogenous form of BCR signaling remains something of an enigma [5]. Both the origin and the effectors of this signal are less understood than antigen-dependent BCR signals, but the signal appears to be important in B cell development and survival of mature, naïve B cells, and is implicated in homeostatic control over the size of the lymphoid compartment between immune responses [4,6]. It is not clear if the same signal is responsible for these several functions; alternatively, the responsible signals might differ from one another either quantitatively or qualitatively. In the present work, an autogenous signal from a mature BCR cooperated with *MYC* to produce a lymphoma that was distinctively different from the tumor produced when the same BCR was subjected to sustained antigenic stimulus. This finding sustains the view that autogenous and antigen-stimulated signaling from the BCR must differ in some way.

In a previous report, introduction of a transgene for the human IgH chain into Eμ-*MYC* mice delayed the onset of tumors and sometimes changed the involved cells to the myeloid and T cell lineages [45]. Using a different strain of Eμ-*MYC* mice [20], we found that a murine BCR accelerated tumorigenesis and did not change the affected lineage. In addition, we confirmed our observations with a second strain of mice that overexpress *MYC* in the B cell lineage (MMTV-rtTA/TRE-*MYC*). We suggest that the different outcomes in the present and previous work may be due to the use of substantially different transgenes, but we have not explored the matter further, because it appears not to bear on our conclusions.

The tumors that arose in the MMTV-rtTA/TRE-*MYC*/BCR^HEL^/sHEL mice displayed sustained dependence upon both the activity of the *MYC* transgene and the stimulus to tumor cells provided by a cognate autoantigen. These findings are in accord with recent reports of diverse mouse models in which the survival and growth of tumors remain dependent upon the genetic lesion that initiated tumorigenesis (for examples, see [46–49]; see [50] for a review). In the present instance, we have demonstrated dependence upon two distinctive tumorigenic influences. *MYC* initiates and is also involved in some level in the maintenance of tumors, whereas antigenic stimulus may sustain cellular proliferation, promote cell survival, or affect cellular differentiation. Moreover, *MYC* can facilitate the effect of autoantigen by breaking immune tolerance in B cells ([51], and see below). The future design of targeted therapies for cancer will probably benefit from the elucidation of such interactions among the steps in tumor progression.

### Immune Tolerance May Have a Role in Lymphomagenesis

We initiated the current work in order to explore the role of antigenic stimulus in the genesis of lymphomas. We used a model in which the bulk of B cells are programmed to respond to a single antigen (HEL), which in turn is also provided as a neo-autoantigen by a transgene. As originally described, mice bearing these two transgenes are anergic for the transgenic autoantigen [25]. So the discovery that the HEL autoantigen could cooperate in tumorigenesis seemed counterintuitive. As reported by us elsewhere [51], however, overexpression of transgenic *MYC* apparently reversibly breaks tolerance in these mice and allows B cells to respond to the HEL autoantigen. Accordingly, the tumors are composed of mature, activated B cells with high-affinity receptors for HEL.

We attribute these findings to the fact that in both B and T cells, the abundant expression of *MYC* can serve as a surrogate for cytokines [51–55]. It has been shown previously that cytokines can override B cell tolerance [56]. Whatever its mechanism, the breach of tolerance by *MYC* in the Eμ-*MYC*/BCR^HEL^/sHEL transgenic mice permits a strong autoantigenic drive of B cell proliferation, and this in turn apparently modifies tumorigenesis by the oncogene.

There is circumstantial evidence that associates broken tolerance with lymphomagenesis in humans. First, the incidence of lymphoid neoplasms is increased in various autoimmune syndromes [57]. For example, individuals with Sjörgen syndrome display a nearly 50-fold increase in the incidence of either diffuse large BCL or follicular BCL [58]. Second, individuals with BL and other forms of non-Hodgkin lymphoma (NHL) frequently have high levels of autoantibodies in their sera [59–64] and develop autoimmune hemolytic anemia [65,66]. In addition, the sequences of the Ig receptors expressed by cells of NHL have been shown to contain mutations that may have arisen during a GC reaction [67]. These mutations may alter receptor specificity, rendering the cells autoreactive. A history of hypermutation in these tumors is also manifested by a high frequency of mutations in alleles of *MYC* that have not been translocated [68]. Given the role of *MYC* in the mouse tumors described here, it might be profitable to explore the effect of other oncogenes on immune tolerance.

### A Mouse Model for BL

We have described two mouse models that develop a lymphoma with a close resemblance to human BL. The similarities include anatomical presentation and other clinical manifestations, histological appearance, and immunophenotype. A particularly striking finding was the unilateral occurrence of jaw tumors in the MMTV-rtTA/TRE-*MYC*/BCR^HEL^/sHEL mice. This manifestation is characteristic of African BL [69], but remains unexplained in both the human and murine setting. In addition, we have shown that the BL-like tumors are composed of cells that have undergone a GC reaction. The evidence supporting this notion includes: expression of cell surface markers that are consistent with a post-GC cell; the presence of class switched immunoglobulins specific to the model autoantigen, HEL; the presence of point mutations in the BCR that are likely the result of somatic mutation that occurs during the GC reaction; and the detection of high levels of mRNA for two GC-associated genes, *Bcl-6* and *AID*. This is the most complete set of parameters yet used to define a mouse model of BL.

Several previous reports have described experimental approaches that might have produced mouse models of BL [20,47,70–73]. Only one of these attempts, however, produced a tumor with substantive resemblances to BL [72]. That model used the control of the Igλ-MYC promoter and enhancer elements to express a mutant form of *MYC* that is found in human NHL. No provision was made for deliberate antigenic stimulation of B cells, but the tumor cells did show evidence of immune selection, in the form of point mutations in the Ig loci. In addition, we have demonstrated the GC origin of the BL tumors in our mouse model, whereas this was not the case for the lymphomas that developed in Igλ-MYC mice [72]. These findings prompted the authors to invoke stimulus by an unidentified antigen in the genesis of the murine tumors. Our work reconstructs such stimulus with a clonal BCR and cognate neo-autoantigen, and demonstrates a contribution of the stimulus to tumorigenesis.

BL appears in two major forms: endemic and sporadic. The endemic form is found mainly in Africa and is characterized by infection with Epstein-Barr virus (EBV) [74,75]. In contrast, an association with EBV infection is found in only about 20% of sporadic BL, but chronic infection with another, as yet unidentified microbe might well figure in the remainder. Viral infection plays no role in the mouse models of BL described here. We presume that the need for such infection has been circumvented by overexpression of the *MYC* transgene, which serves as a surrogate for the translocations that are a hallmark of human BL and are thought to occur subsequent to initiation of tumorigenesis by EBV or another agent [76].

With few exceptions, the tumor cells of BL have been described as monoclonal [77–80]. In contrast, the tumor cells in the two animal models for BL described here are multiclonal. How might we explain this distinction? The human tumor presumably arises from a series of rare events, each amplified by clonal selection ([81]; reviewed in [82]). The cumulative rarity in this sequence of events dictates that the eventual tumor is likely to be the product of a single clonal lineage. In contrast, the experimental model described here provides at least two potentially tumorigenic influences that are ubiquitous in the B cell lineage of the transgenic animals: overexpression of *MYC* and stimulus by an autoantigen. Thus, a vast population of cells may be predisposed to tumor progression. Indeed, it is remarkable that the resulting tumors are composed of only a finite number of clones, suggesting the occurrence of clonal selection for tumorigenic events beyond those imposed experimentally. The results contrast sharply with the innumerable clones that proliferate to produce a relatively indolent disease in MRL*^lpr/lpr^* mice, a proliferation that is itself driven by autoimmunity .

A variety of circumstantial evidence has implicated antigenic stimulus in the genesis of BL [83]. First, chronic infection with malaria in Africa is associated with an increased incidence of BL and accelerated progression of the disease [84,85]. Second, the possibility of sustained antigenic stimulus is raised by the mature, activated immunophenotype characteristic of BL cells [86]. Third, the sequences for the immunoglobulin molecules in many NHL, including BL, bear somatic mutations of the sort that normally arise during the process of affinity maturation [8–14] If antigenic stimulation does play a role in the genesis of human BL, it would be in cooperation with *MYC*, whose activation by chromosomal translocation is a general feature of the tumor [87]. Our results with mouse models suggest that the hypothetical role of antigenic stimulus in the pathogenesis of BL should be pursued further.

### The Response of Murine BCLs to Immunosuppressants

We have shown that antigenic stimulus can apparently contribute to the establishment and maintenance of B-cell lymphomas in mice. The tumors that arose in the Eμ-*MYC*/BCR^HEL^/sHEL and MMTV-rtTA/TRE-*MYC*/BCR^HEL^/sHEL mice expressed a neo-autoantigen (sHEL), which provided an autoimmune stimulus to the tumor cells. This in turn allowed the tumors to become self-sufficient and retain continuous cognate antigen stimulation upon transplantation. This notion was further supported by experiments in which the signaling components of the BCR were genetically disrupted with shRNAs.

The requirement for antigen in turn suggested that the tumors might respond to pharmacological interruption of signaling from the BCR. This proved to be the case: treatment with cyclosporin, FK506, or rapamycin elicited prolonged remissions. These results conform to the view that antigen-induced signaling from the BCR was involved in the genesis of the tumors. We recognize, however, that none of the immunosuppressants acts exclusively on signaling from the BCR and, as a result, each might inhibit tumors by a different means [88–90]. For example, it has been reported that rapamycin can inhibit angiogenesis in certain solid tumors [91], whereas other immunosuppressants failed to demonstrate this inhibition. In contrast, rapamycin, cyclosporin, and FK506 all inhibit signaling from the BCR [92–94], and all three elicited remissions of Eμ-*MYC*/BCR^HEL^/sHEL and MMTV-rtTA/TRE-*MYC*/BCR^HEL^/sHEL tumors. Moreover, this uniform effect of the three immunosuppressants did not extend to tumors in which antigen stimulus ostensibly played no role (Eμ-*MYC* and Eμ-*MYC*/BCR^HEL^).

We conclude that the therapeutic effects of immunosuppressants reported here for the BL model probably reflect the role of antigen-induced signaling from the BCR in the pathogenesis of the tumor. Tactics that interrupt expression of signaling from the BCR might also be useful in the treatment of human lymphomas that express the receptor.

## Methods

### Transgenic mice and transplantation of tumors.

Mice carrying the Eμ-*MYC* transgene have been described previously [20] and were obtained from the Jackson Laboratory. These mice express *MYC* in a B cell–specific manner, beginning at the Pre/Pro-B cell stage. The TRE-*MYC* and MMTV-rtTA mice have been described previously [21,23]. We crossbred these strains to combine the two transgenes in a single strain (MMTV-rtTA/TRE-*MYC),* in which the B cell–specific expression of the *MYC* transgene can be repressed by the administration of tetracycline or doxycycline. We also used both BCR^HEL^ mice, which express a pre-rearranged murine BCR from the endogenous immunoglobulin promoter, and sHEL mice, which ubiquitously express a transgene for the soluble form of soluble HEL under the control of the metallothionein promoter. These two strains have been described previously [25] and were generously provided by Jason Cyster (University of California, San Francisco). We also used a strain of mice in which a previously rearranged IgH VDJ sequence was knocked into the IgH locu [95]. When used in combination with another strain, encoding an IgL transgene (Lt-tg), those bigenic mice generate about 30% HEL-specific B cells, as previously describe[95]5]. Those two strains of mice were kindly provided by Jason Cyster, at UCSF. All transgenic mouse lines were maintained on a C57/BL6 background, and were genotyped by PCR as previously described [20,25,96]. All animals were maintained in accordance with the guidelines of the Committee on Animal Research at the University of California, San Francisco, and the National Research Council.

Adoptive transfers of cells and transplantation of tumors were done by injecting 10^6^ cells intravenously (unless otherwise indicated) into syngeneic (C57/BL6) females ranging in age from 4–6 wk. For the experiments that involved tumor cells obtained from MMTV-rtTA/TRE-*MYC*/BCR^HEL^/sHEL tumors, the recipient mice were sublethally irradiated (450 rads) in order to overcome some remaining allogeneic differences between the two strains.

### Assessment of tumorigenesis.

The emergence of tumors was followed in three ways: (i) physical examination of living animals and necropsy of deceased animals, particularly to detect enlargement of lymphoid organs and viscera; (ii) counting the total number of cells in organs; and (iii) the specific enumeration of B cells carrying cell-surface receptor for the antigen HEL. Three pairs of lymph nodes were collected each time (two inguinal, two axillary, and two brachial lymph nodes). These lymph nodes were pooled and processed into single-cell suspensions. Spleens and thymii were also collected and used to generate single-cell suspensions. Each spleen or thymus was individually ground on a 60-μm wire mesh screen (Sigma). The red blood cells were lysed in TAC buffer (0.017 M Tris, pH 7.65, and 0.135 M NH_4_Cl), as previously described [95], and the resulting pellets were resuspended in complete lymphocyte media, which consists of RPMI1640 + 10% heat inactivated fetal calf serum, supplemented with L-glutamine, penicillin/streptomycin, nonessential amino acids, 2 mM HEPES, 2mM sodium pyruvate, and 10 mM β-mercaptoeathanol (all obtained from Invitrogen). Single-cell suspensions were counted with a Coulter counter (Coulter Diagnostics). The percentage of viable cells was determined by uptake of 7-aminoactinomycin D (7AAD) and flow cytometry. The values for total cell numbers were used to derive the number of viable cells by multiplying percentage of viable cells (obtained from the 7AAD analysis) by the total number of cells (obtained from the Coulter counter analysis), and dividing by 100. These measurements were compared with microscopic counting of trypan-blue excluding cells in a hemocytometer.

To determine the number of B cells carrying the BCR^HEL^ transgene, single-cell suspensions were stained with antibodies to B220 and IgM^a^ (both obtained from Pharmingen Laboratories), followed by flow cytometric analysis. The number of BCR^HEL^+ B cells was determined by multiplying the percentage of B220+/IgM^a^+ cells (obtained from the FACS analysis) by the number of total viable cells and dividing by 100. These values were compared to stains performed using a pan-specific antibody to IgM (Pharmingen Laboratories). This approach was used to determine the number of BCR^HEL^ expressing cells in all the cases where the mice were on a C57/BL6 background, where the allotype expressed is normally IgM^b^. For the mice in which the genetic background was mixed (all the experiments that involved the MMTV-rtTA/TRE-*MYC* transgenes), the number of BCR^HEL^-expressing cells was determined by HEL binding. Single-cell suspensions were incubated with HEL (1 mg/ml, obtained from Sigma) in FACS buffer. These cells were washed and incubated with Hy9-biotin, an HEL-specific monoclonal antibody (kindly provided by Jason Cyster, UCSF), followed by streptavidin-PE and B220-FITC (both obtained from Pharmingen Laboratories).

###  Phenotypic analysis of cells.

The surface phenotype of cells present in the lymphoid organs of normal and tumor-bearing mice was analyzed by flow cytometry. Single cell suspensions were prepared from the lymph nodes, spleens, thymus, and bone marrow. The cell suspensions were incubated with 1:50 dilutions of antibodies on ice for 30 min, and were then washed in FACS buffer (1% BSA in PBS + 0.05% sodium azide) and fixed in PBS containing 1% paraformaldehyde. Cells were stained with antibodies to one or more of the following markers: B220, Thy1.2, Mac-1, IgM (pan), IgM^a^, IgM^b^, IgD (pan) and IgD^a^, CD4, CD5, CD8, CD19, CD21, CD23, CD25, CD44, CD62L, CD69, CD80, and/or CD86 (all obtained from BD-Pharmingen). Binding of HEL to B cells was assessed by incubating cell suspensions with 1 mg/ml HEL (Sigma) in FACS buffer. The cells were then washed and incubated with Hy9-biotin, followed by Streptavidin-PE (BD-Pharmingen).

### Molecular analysis of tumor clonality.

To determine the clonal composition of the tumors, we adapted a protocol that has been described previously [97]. Genomic DNA was extracted from 10^6^ cells (from either spleen or lymph nodes) using the Quiagen genomic DNA mini-kit (Quiagen), following the manufacturer's specifications. 200 ng of genomic DNA was used for a nested PCR reaction. The first reaction consisted of 5 μl of 10X Taq buffer (Gibco/Invitrogen), 4 μl of 50 mM MgCl_2_, 2.5 ng of V_H_-specific primer, 2.5 ng of J_H-_specific primer, 2.5 nM dNTPs (Roche Diagnostics) and 2.5 U of Taq polymerase (Roche Diagnostics) and distilled-deionized water to a final volume of 50 μl. The reactions were placed in a thermal cycler (MJ-Research) and subjected to a PCR cycle as previously described [97]. A sample of 2 μl from the first reaction was used as a template for the second reaction of the nested PCR. This reaction was conducted as the first one, except that the primer pairs encoded sequences within the initial set used earlier. The sequences for all the primers used have been previously described [96]. The PCR reaction products were fractionated in a 2% agarose/TAE gel, stained with ethidium bromide. Some of the PCR products were cloned using a TOPO-TA cloning kit (Invitrogen Laboratories), following manufacturer's specifications, then sequenced using the Big Dye terminator cycle sequencing kit (Applied Biosystems), following manufacturer's specification, at the UCSF General Clinical Research Centers core facility.

### Disruption of gene expression by shRNA in tumor cells.

We used the Reynolds algorithm [98] and pSICO oligomaker software [35] in order to design the optimal target shRNA sequences. Those core 19-mer sequences were incorporated into the oligonucleotides designed to contain the stem and loop portions of the shRNA to be cloned into the vector pLL3.7 [36]. Cloning was performed as previously described [36]. The specific sequences we used to disrupt expression of murine Igα, Igβ or the control (firefly luciferase) have been described elsewhere [95].

All constructs presented here were initially validated for their ability to specifically knock down the expression levels of the protein of interest by transduction of two BCLs that we generated from the mouse models presented here. The two types of B cell lines we generated were derived from Eμ-*MYC*/BCR^HEL^ (DBL: double transgenic B cell lymphoma) and Eμ-*MYC*/BCR^HEL^/sHEL (TBL: triply transgenic B cell lymphoma) primary murine tumors. DBL and TBL cell lines were generated by passive selection from Eμ-*MYC*/BCR^HEL^ and Eμ-*MYC*/BCR^HEL^/sHEL primary murine tumors, respectively. Tumor B cell lines were grown in C10 (RPMI, 10% FBS (HyClone), 2 mM L-glutamine (Invitrogen) 100 units/ml penicillin G and streptomycin sulfate (Invitrogen), 10 mM HEPES, 0.1 mM MEM non-essential amino acids (Invitrogen), and 0.55 mM β-mercaptoethanol (Invitrogen)). The GFP+ fraction of the transduced cell population was used for either Western blotting or flow cytometric analysis of protein expression.

To generate infectious viral particles, we used 293FT cells as a packaging system that had been previously described [99]. 293FT cells were grown in D10 media (DMEM, 10% FBS (HyClone), 2 mM L-glutamine (Invitrogen), 100 units/ml penicillin and streptomycin (Invitrogen), and 0.1 mM MEM non-essential amino acids (Invitrogen). 293FT cells grown to 60% confluency in 60-mm dishes were transfected with 5 μg pLL3.7, 3.3 μg pMDLg/pRRE, 1.3μg pRSV-REV, and 1.9μg pMD.G [99] overnight using calcium-phosphate methods, as described previously [36]. Media were replaced with 4.5 ml fresh D10 the following morning.

Infections were performed as previously described [35]. Briefly, supernatants from 293FT cells were harvested 2 d after transfection and replaced with fresh D10. Viral supernatants were passed through a 0.45 μm filter and brought to a final concentration of 8 μg/ml polybrene and 10 mM HEPES, pH 7.4. These supernatants were overlayed on 2 × 10^5^ DBL and TBL cells and spun at 2,000 rpm for 1 h at 25 °C. Following spinfection, viral supernatants were removed and replaced with fresh C10. Infections were repeated the following day (3 d after transfection).

For the in vitro assays used to determine whether the disruption of a gene product affected the competitiveness of a tumor cell, we used a mixed of transduced and nontransduced cells. The efficiency of viral transduction of DBL and TBL cell lines was initially evaluated 48 h after the second spin infection. We subsequently tracked infected cells for two weeks post-infection. For analysis, we normalized all infection efficiencies to the 48-h time point, and further normalized each day to the infection rate for the pLL3.7 alone.

### Therapeutic trials.

Groups of six mice were used for each of the experimental protocols. Four mice bearing transplanted tumors, and two age- and sex-matched wild-type mice were treated with the same drug and equal frequency. The transplant-recipient mice were held until tumors became clinically apparent (approximately 100 d for the Eμ-*MYC* tumors, 58 d for the Eμ-*MYC*/BCR^HEL^ tumors, 21 d for the Eμ-*MYC*/BCR^HEL^/sHEL tumors, and 14 d for the MMTV-rtTA/TRE-*MYC*/BCR^HEL^/sHEL tumors). The mice then received daily injections of the indicated agents for 7 d. Mice were either euthanized 24 h after the last injection of drug, or held indefinitely to ascertain survival. Lymph nodes, spleens and bone marrows were collected and processed to generate single-cell suspensions. The cells were counted as described above. An aliquot from the cell suspensions was stained with antibodies for B220, Thy 1.2, Mac-1, IgM^a^, B7–2, and CD69, in order to determine the proportion of B cells, T cells, and myeloid cells, as well as the activation status of the HEL-reactive B cells. Treatments were performed with cyclosporin A (Bedford Laboratories) (2 mg/kg/day), FK506 (Prograf, Fujisawa Healthcare) (2 mg/kg/day), rapamycin (Biomol) (2 mg/kg/day), and cyclophosphamide (Bristol-Myers Squibb) (1 mg/kg/day). The therapeutic agents were suspended in PBS, sterilized by filtration through a 0.22μm membrane, and administered intravenously through the tail vein.

### Tissue processing and histology.

Normal and tumor tissues were fixed in 10% formalin and embedded in paraffin. Sections (4μm) were stained with hematoxylin-eosin. Images were acquired with a CCD camera mounted on a phase-contrast microscope.

## Supporting Information

Figure S1 Immunophenotype of B Cell Tumors Flow cytometric analysis was performed on spleen cells from a wild-type mouse (orange trace), a tumor-bearing Eμ-*MYC*/BCR^HEL^ mouse (blue trace), or a tumor-bearing Eμ-*MYC*/BCR^HEL^/sHEL mouse (pink trace), and cells from a jaw tumor in an MMTV-rtTA/TRE-*MYC*/BCR^HEL^/sHEL mouse (green trace). Staining for the indicated surface markers was compared to unstained spleen cells from wild-type mice (filled purple trace).
(2.28 MB TIF)

Figure S2 Clonality of Tumors Genomic DNA was analyzed for V_H_ to DJ_H_ rearrangements as described in the Methods section. We examined rearrangements of 16 different combinations of four V region genes (lanes numbered 1–4, as follows: 1 corresponds to 36–6, 2 to 81X, 3 to Q-52, and 4 to J558). These were tested in combination with the four J_H_ genes listed in the figure (J_H_1–4). The arrows in the lower left corners of the panels indicate the PCR products that resulted from amplification of the germ line configuration. All of the rearranged VDJ_H_ products migrated more slowly in the gel. The data are representative of three different matched pairs of primary and transplanted tumors, for each tumor type.
(A) Wild-type spleen.
(B) Spleen cells from a 6 month old MRL*^lpr/lpr^* mouse with lymphoproliferative disease.
(C) Spleen tumor from an Eμ-*MYC*/BCR^HEL^ mouse.
(D) Spleen cells from a mouse 60 d after receiving a transplant of the cells analyzed in (C).
(E) Spleen tumor from an Eμ-*MYC*/BCR^HEL^/sHEL mouse.
(F) Spleen cells from a mouse 23 d after receiving a transplant of the cells analyzed in (E).
(G) Jaw tumor from an MMTV-rtTA/TRE-*MYC*/BCR^HEL^/sHEL mouse.
(H) Spleen cells from a mouse 14 d after receiving a transplant of cells analyzed in (G).
(4.27 MB TIF)

Figure S3 Regulation of TRE-*MYC* Transgene Expression by Docycycline in B Cells Obtained from MMTV-rtTA/TRE-*MYC*/BCR^HEL^ Transgenic Mice Splenic B cells were obtained from said mice, activated in vitro for 3 d with antibodies to IgM and CD40, in the presence or absence of docycycline. Cells were then lysed and subjected to SDS-PAGE electrophoresis and western blot analysis. The antibody used to detect human *MYC*, encoded by the transgene was 9E10.
(995 KB TIF)

Figure S4 Murine Burkitt-Like Tumors Are Composed of Post-GC B Cells *Post-GC nature of Burkitt-like tumors*: The large BCLs in humans, including BL, are typically derived from post-GC B cells. Based on histological appearance and cell-surface markers, we suggested that the tumors arising in Eμ-*MYC*/BCR^HEL^/sHEL and MMTV-rtTA/TRE-*MYC*/BCR^HEL^/sHEL mice resembled BL (see Results section). To further authenticate the resemblance, we sought evidence that the mouse tumors were derived from post-GC B cells. We used three criteria to define whether the cells derived from the murine BCLs in our models have undergone a GC reaction. First, evidence of immunoglobulin class switching; second, hypermutation in the nucleic acid sequence encoding for the BCR hypervariable regions expressed by the tumor cells; and third, expression of genes associated with the GC process.
We could not explore class switching and somatic mutation with the mouse strains used to this point, because the BCR^HEL^ transgene was not controlled by the internal elements of the IgH locus, was configured to generate mature IgM and IgD isotypes, and could not undergo further class switching. Instead, we obtained two additional genetically modified mouse strains that would enable this analysis with a defined antigenic specificity: one in which the hypervariable region for the IgH specific to HEL had been recombined into the corresponding site of the IgH locus, named VDJki [95], and another that harbored a transgene encoding the IgL that would normally pair with the corresponding IgH in the HEL-specific hybridoma from which the hypervariable region was cloned, named Lt-tg [95]. Those two alleles have previously been shown to give rise to HEL-specific B cells that can undergo a GC reaction, as determined by their ability to produce HEL-specific antibodies that had class-switched [95]. The key advantage of using the VDJki/Lt-tg mice to generate HEL-specific B-cells over BCR^HEL^ transgenic mice is the ability of the former to undergo somatic mutations and class switching in a GC-dependent manner, since the HEL-specific components were integrated into the normal locus [95].
In order to facilitate this strategy, we created a strain of mice (Eμ-*MYC*/VDJki/Lt-tg/sHEL) that incorporated the knock-in allele for generating HEL-specific B cells, and used this strain to examine whether the cells that composed the resulting BL-like tumors had been selected in the GC. The mice developed BCLs at about 50–64 d after birth, with complete penetrance (unpublished data). The resulting tumors were present in the lymph nodes, spleen, thymus, and bone marrow, and also infiltrated other organs, including the liver, lungs, kidney, and central nervous system (unpublished data). The tumor cells were B220+, CD19+, IgM+, CD69+, B7–2+, CD21+, CD23-, and PNA+, consistent with a mature, activated, post-GC phenotype, and identical to what we had observed previously with the Eμ-*MYC*/BCR^HEL^/sHEL mice (Table 1). In addition, the tumors that arose from Eμ-*MYC*/VDJki/Lt-tg/sHEL mice displayed the characteristic “starry sky” histopathogy, and could be readily transplanted into unmanipulated, syngeneic recipient mice (unpublished data). We also tested the dependence of the tumors that arose in Eμ-*MYC*/VDJki/Lt-tg/sHEL mice upon continuous BCR-derived signals. We used the shRNA-mediated knockdown approach to target Igα and Igβ, as shown in Figure 4. We observed that the tumors that developed in Eμ-*MYC*/VDJki/Lt-tg/sHEL mice required Igα or Igβ expression for their maintenance in vitro, suggesting that they depend upon continuous antigenic stimulation (unpublished data).
To test whether the cells that compose the tumors obtained from the Eμ-*MYC*/IgH129ki/Lt-tg/sHEL mice had undergone a GC reaction, we examined three criteria: the presence of HEL-specific immunoglobulins in the sera of the tumor-bearing mice that are of different classes (IgM, IgG, and IgA); the presence of point mutations in the VDJ joint sequences of the IgH129ki allele, consistent with somatic mutations that occur in the germinal center during the process of affinity maturation; and the expression of two GC-associated genes, *Bcl-6* and activation-induced cytidine deaminase (*AID*).
The first difference that we observed between the tumors that developed in Eμ-*MYC*/IgH129ki/Lt-tg/sHEL mice and those from Eμ-*MYC*/BCR^HEL^/sHEL mice was the secretion of additional Ig types (A). In addition to HEL-specific IgM (found also in the tumors expressing the BCR^HEL^ transgene), we also detected HEL-specific IgG1, IgG2, IgG3, and IgA, as expected from the class switching that occurs in the GC reaction. In addition, not all the tumor-bearing mice had all Ig isotypes, suggesting clonal differences among the different tumors.
We amplified the VDJ joint sequence from eight tumors obtained from Eμ-*MYC*/IgH129ki/Lt-tg/sHEL mice by PCR-based methods, then cloned the amplified products and sequenced the contents obtained from 120 independent colonies. Every sequence obtained from those tumors contained mutations in the VDJ sequence of the IgH129ki allele. Moreover, we found several clones that contained the identical patterns of mutations (B). By contrast, we noticed one mutation among 20 alleles we sequenced from a normal, healthy IgH129ki mouse, which we attribute to a PCR error.
While the class switching and somatic mutation data should suffice to formally prove that the BL-like tumors are composed of post-GC cells, we also sought to determine whether those cells express transcripts of two genes that are normally associated with the GC reaction. Accordingly, we developed assays for real-time, semi-quantitative RT-PCR for *Bcl-6* and *AID*. *Bcl-6* is highly expressed in human DLBL, and a loss of function mutation in mice was shown to lead to defective formation of GC [100]. *AID* was shown to be critical for the processes of class-switch recombination and somatic mutation that are carried out during a GC reaction [101]. Our results show that 8/8 tumors expressed high levels of *Bcl-6* transcripts, and 5/8 tumors expressed high levels of *AID* mRNA relative to normal splenic B cells (C). In addition, we were also able to detect high levels of *Bcl-6* mRNA in tumors that developed in Eμ-*MYC*/BCR^HEL^/sHEL mice, although we were not able to detect *AID* transcripts in those tumors (*n* = 10) (C). We did not observe any detectable levels of mRNAs for either *AID* or *Bcl-6* in the tumors that developed in Eμ-*MYC*/BCR^HEL^ mice (C).
These data allow us to conclude that the *MYC*-driven, antigen-dependent BCLs in the Eμ-*MYC*/IgH129ki/Lt-tg/sHEL mice were composed of post-GC cells, in accord with their other resemblances to BL. Thus, by diverse measures, the tumors in Eμ-*MYC*/IgH129ki/Lt-tg/sHEL mice provide a reasonable facsimile of BL. In addition, the conclusions obtained from these studies likely extend to our additional mouse models of BL.
(A) Analysis of class-switching in HEL-specific BCLs. Groups of eight mice for each genotype described were used for these assays. The sera samples were examined for the presence of HEL-specific immunoglobulins using a solid-phase either bled at 8 wk of age (all of the control groups), or upon presenting of clinical signs of disease (for Eμ-*MYC*/BCR^HEL^/sHEL and VDJIki/Lt-tg/BCR^HEL^/sHEL mice). ELISA assay. Sera were obtained from groups of eight mice of the specified genotypes and assayed in triplicate by ELISA against HEL, using isotype-specific secondary antibodies. The sera were obtained from wild-type mice (column 1), naïve BCR^HEL^ mice (column 2), BCR^HEL^ that were immunized with HEL emulsified in complete Freund's adjuvant 14 d prior to bleeding (column 3), naïve VDJki/Lt-Tg mice (column 4), VDJki/Lt-Tg mice that were immunized with HEL emulsified in complete Freund's adjuvant 14 d prior to bleeding (column 5), tumor-bearing Eμ-*MYC*/BCR^HEL^/sHEL mice (column 6), or tumor-bearing VDJIki/Lt-tg/BCR^HEL^/sHEL mice (column 7). The results shown here are from one representative experiment of three.
(B) Analysis of somatic mutations in the immunoglobulin joint sequence. We PCR-amplified and sequenced the VDJ-joint sequence of the HEL-specific BCR in tumor-bearing mice in order to determine whether GC-associated somatic mutation was evident in BL tumors. Cells were obtained from tumor-bearing Eμ-*MYC*/VDJki/Lt-Tg/sHEL mice and used to obtain genomic DNA. The DNAs were used to perform a PCR reaction with primers surrounding the IgH VDJ joint region used for generating the knock-in mutation. PCR products were cloned into TOPO cloning vectors and sequenced. The bold, underlined letters in the sequence show mutations found in the tumors, as opposed to the sequences obtained from a VDJki/Lt-Tg mouse (20 clones), presented at the bottom of the table. We only detected one point mutation among the 20 clones that we sequenced from normal VDJki/Lt-tg B-cells.
(C) Detection of mRNAs encoding Bcl-6 and AID in tumors obtained from Eμ-*MYC*/VDJki/Lt-tg/sHEL transgenic mice. Cells were obtained from either normal VDJki/Lt-tg mice (column 1), tumor-bearing Eμ-MYC mice (column 2), or tumor-bearing Eμ-*MYC*/VDJki/Lt-Tg/sHEL mice (columns 3–10). All cell suspensions were depleted from their T cells, and used to generate RNA with a Trizol reagent. These RNAs were used to generate cDNAs in vitro with random primers. The resulting cDNAs were then used for real-time, semiquantitative RT-PCR, using SYBR green for fluorescent detection. We used oligonucleotides specific for AID or Bcl-6, as previously reported [102, 103]. The values presented were standardized to the levels of two housekeeping genes (*GAPDH* and *L32*). The results presented here are from one assay representative of three independent assays.
(4.05 MB TIF)

Table S1 Comparison of Human and Mouse Tumors (37 KB DOC)

## Abbreviations

BCL: B cell lymphoma
B-CLL: B cell lymphocytic leukemia or lymphoma
BCR: B cell receptor
BL: Burkitt lymphoma
DLBCL: diffuse large B cell lymphoma
GC: germinal center
HEL: hen egg lysozyme
NHL: non-Hodgkin lymphoma
