# Supplementary figures and images for: The B Cell Antigen Receptor and Overexpression of MYC Can Cooperate in the Genesis of B Cell Lymphomas
Source: PLoS Biol. 2008 Jun 24;6(6):e152. doi: 10.1371/journal.pbio.0060152 (PMC2435152; doi:10.1371/journal.pbio.0060152)

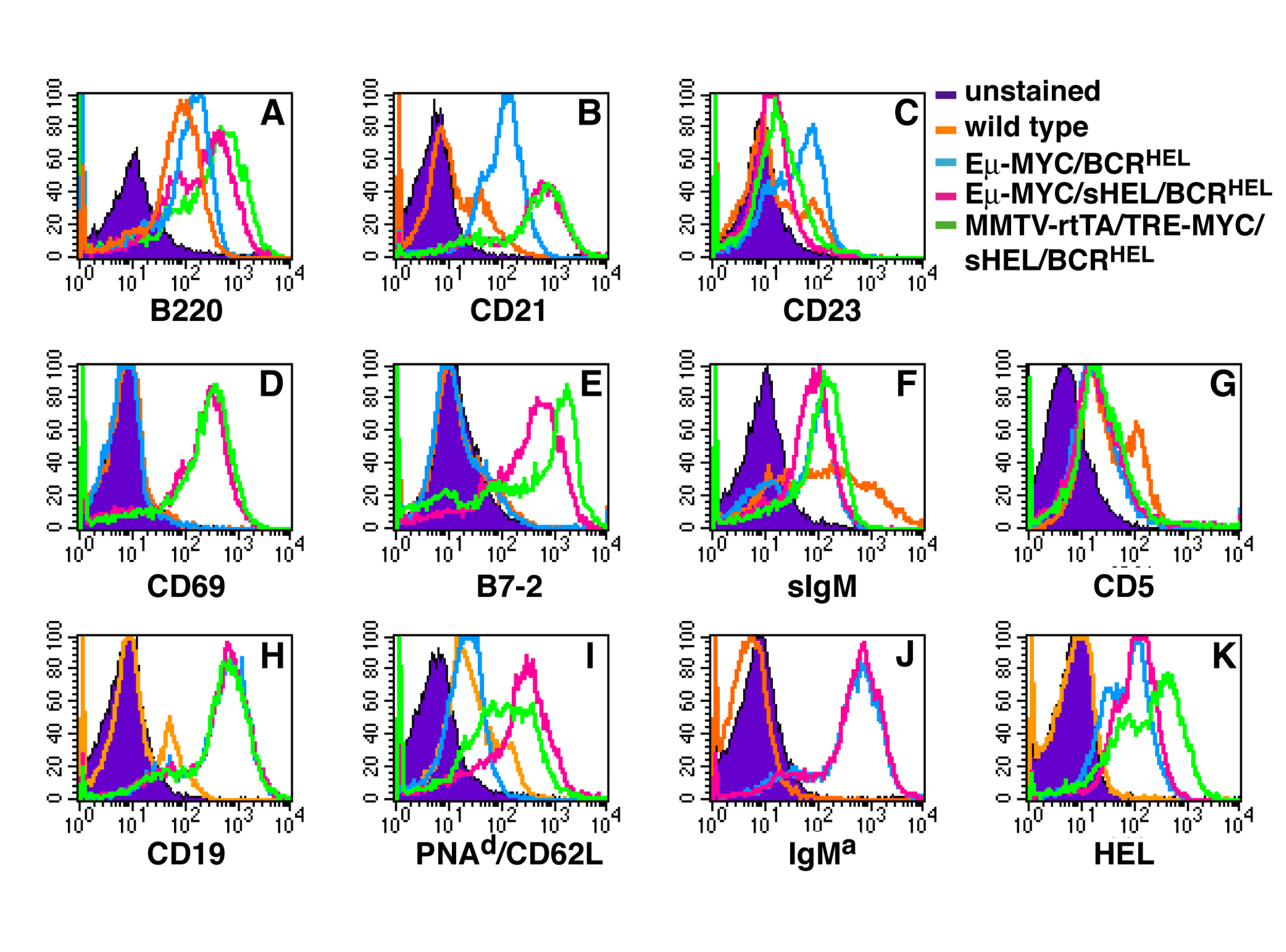

Supplement: Figure S1 — Flow cytometric analysis was performed on spleen cells from a wild-type mouse (orange trace), a tumor-bearing Eμ-MYC/BCRHEL mouse (blue trace), or a tumor-bearing Eμ-MYC/BCRHEL/sHEL mouse (pink trace), and cells from a jaw tumor in an MMTV-rtTA/TRE-MYC/BCRHEL/sHEL mouse (green trace). Staining for the indicated surface markers was compared to unstained spleen cells from wild-type mice (filled purple trace). (2.28 MB TIF) [file pbio.0060152.sg001.tif]

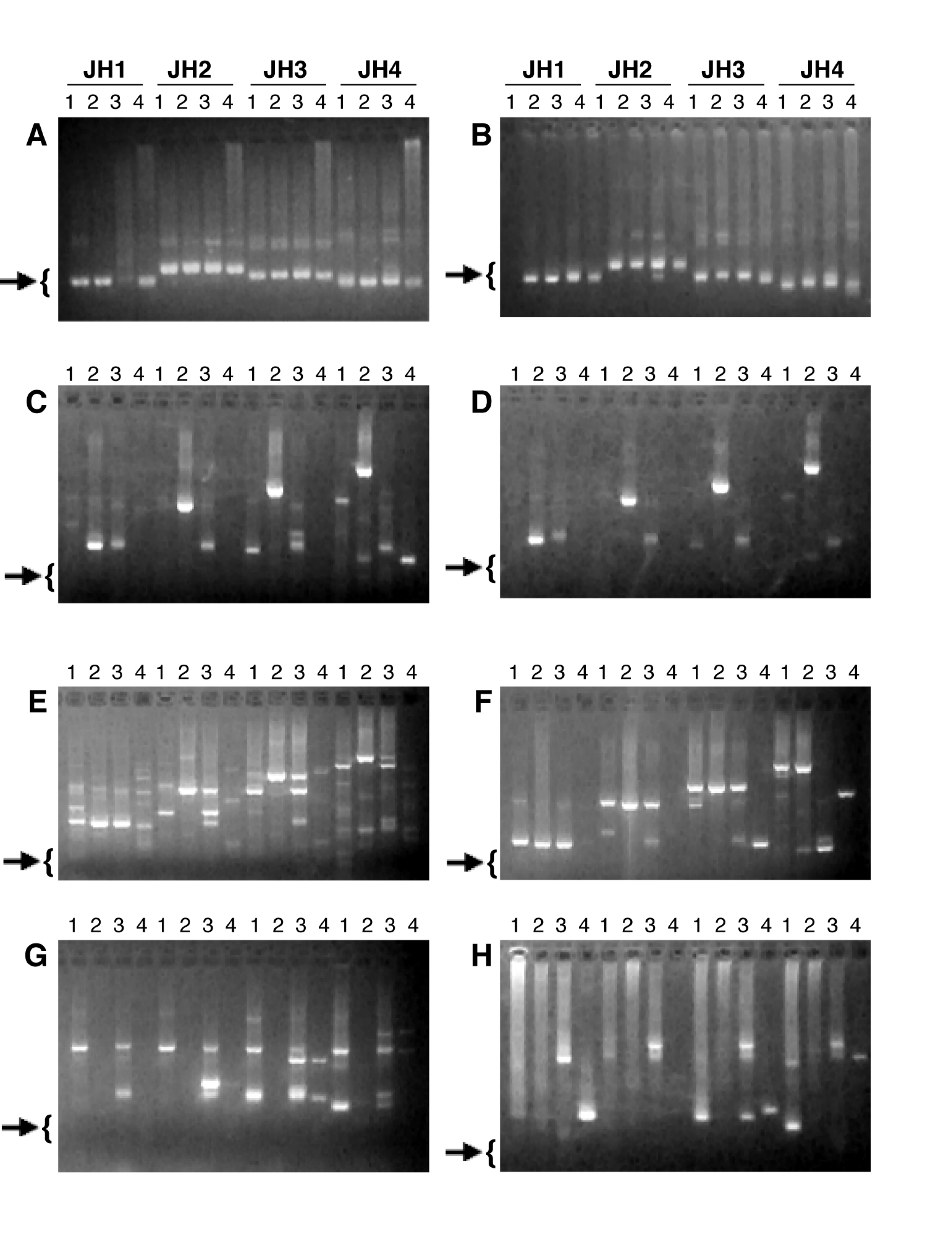

Supplement: Figure S2 — Genomic DNA was analyzed for VH to DJH rearrangements as described in the Methods section. We examined rearrangements of 16 different combinations of four V region genes (lanes numbered 1–4, as follows: 1 corresponds to 36–6, 2 to 81X, 3 to Q-52, and 4 to J558). These were tested in combination with the four JH genes listed in the figure (JH1–4). The arrows in the lower left corners of the panels indicate the PCR products that resulted from amplification of the germ line configuration. All of the rearranged VDJH products migrated more slowly in the gel. The data are representative of three different matched pairs of primary and transplanted tumors, for each tumor type. (A) Wild-type spleen. (B) Spleen cells from a 6 month old MRLlpr/lpr mouse with lymphoproliferative disease. (C) Spleen tumor from an Eμ-MYC/BCRHEL mouse. (D) Spleen cells from a mouse 60 d after receiving a transplant of the cells analyzed in (C). (E) Spleen tumor from an Eμ-MYC/BCRHEL/sHEL mouse. (F) Spleen cells from a mouse 23 d after receiving a transplant of the cells analyzed in (E). (G) Jaw tumor from an MMTV-rtTA/TRE-MYC/BCRHEL/sHEL mouse. (H) Spleen cells from a mouse 14 d after receiving a transplant of cells analyzed in (G). (4.27 MB TIF) [file pbio.0060152.sg002.tif]

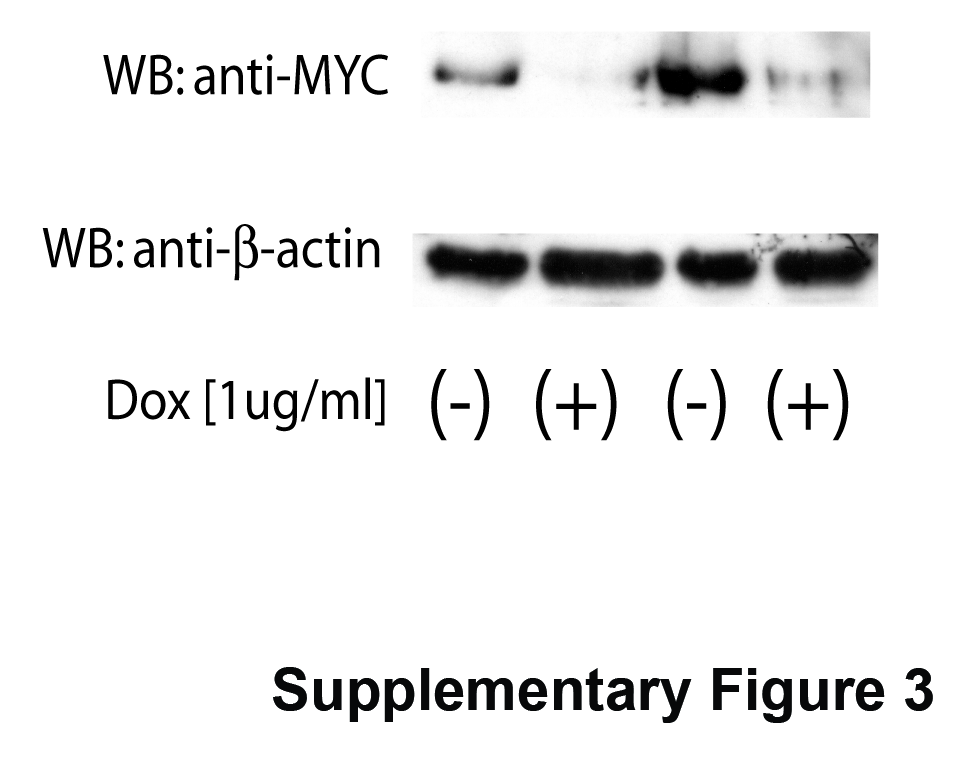

Supplement: Figure S3 — Splenic B cells were obtained from said mice, activated in vitro for 3 d with antibodies to IgM and CD40, in the presence or absence of docycycline. Cells were then lysed and subjected to SDS-PAGE electrophoresis and western blot analysis. The antibody used to detect human MYC, encoded by the transgene was 9E10. (995 KB TIF) [file pbio.0060152.sg003.tif]

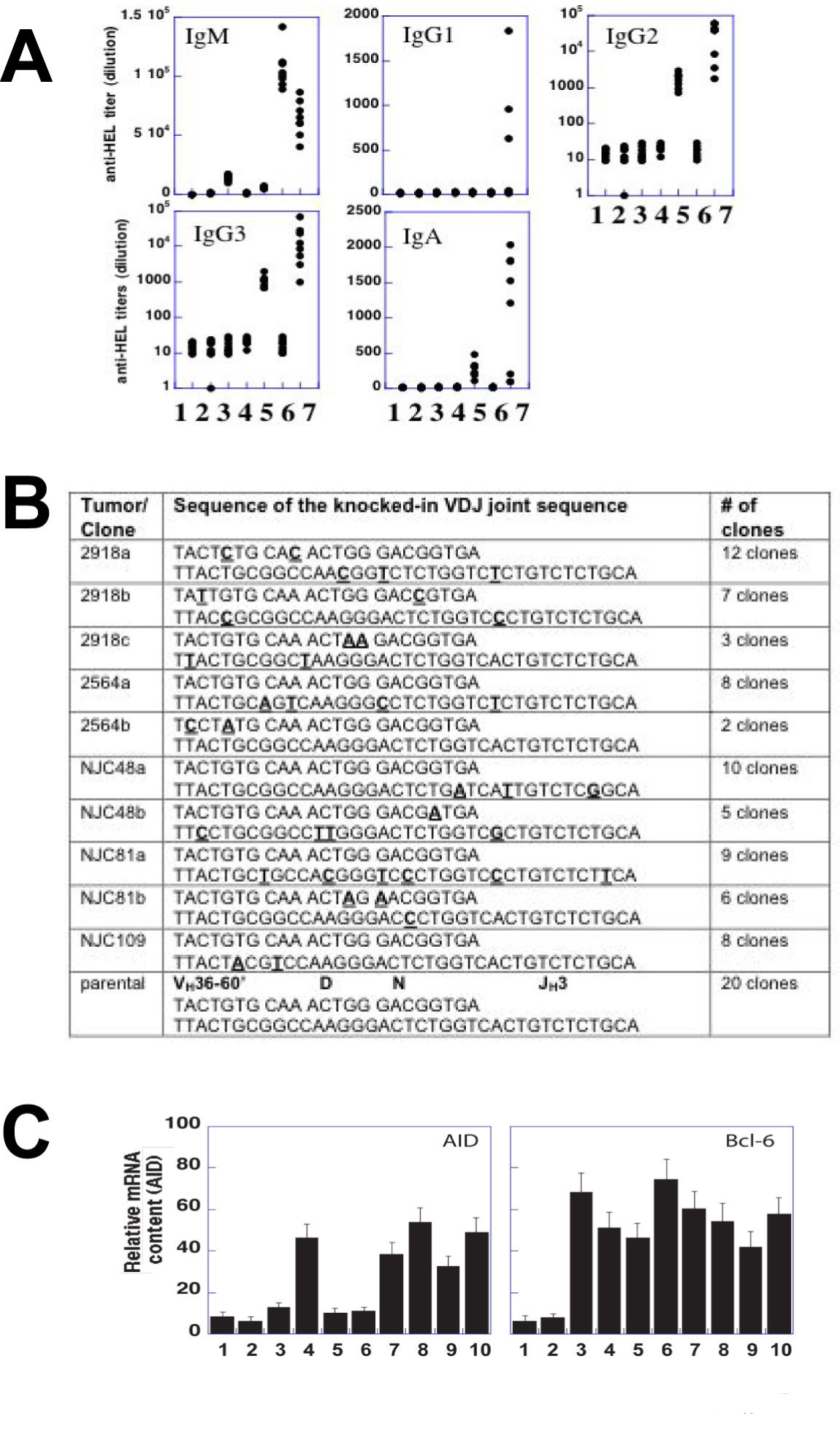

Supplement: Figure S4 — Post-GC nature of Burkitt-like tumors: The large BCLs in humans, including BL, are typically derived from post-GC B cells. Based on histological appearance and cell-surface markers, we suggested that the tumors arising in Eμ-MYC/BCRHEL/sHEL and MMTV-rtTA/TRE-MYC/BCRHEL/sHEL mice resembled BL (see Results section). To further authenticate the resemblance, we sought evidence that the mouse tumors were derived from post-GC B cells. We used three criteria to define whether the cells derived from the murine BCLs in our models have undergone a GC reaction. First, evidence of immunoglobulin class switching; second, hypermutation in the nucleic acid sequence encoding for the BCR hypervariable regions expressed by the tumor cells; and third, expression of genes associated with the GC process. We could not explore class switching and somatic mutation with the mouse strains used to this point, because the BCRHEL transgene was not controlled by the internal elements of the IgH locus, was configured to generate mature IgM and IgD isotypes, and could not undergo further class switching. Instead, we obtained two additional genetically modified mouse strains that would enable this analysis with a defined antigenic specificity: one in which the hypervariable region for the IgH specific to HEL had been recombined into the corresponding site of the IgH locus, named VDJki [95], and another that harbored a transgene encoding the IgL that would normally pair with the corresponding IgH in the HEL-specific hybridoma from which the hypervariable region was cloned, named Lt-tg [95]. Those two alleles have previously been shown to give rise to HEL-specific B cells that can undergo a GC reaction, as determined by their ability to produce HEL-specific antibodies that had class-switched [95]. The key advantage of using the VDJki/Lt-tg mice to generate HEL-specific B-cells over BCRHEL transgenic mice is the ability of the former to undergo somatic mutations and class switching in a GC-dependent m [file pbio.0060152.sg004.tif]
