# Supplementary material for: The B Cell Antigen Receptor and Overexpression of MYC Can Cooperate in the Genesis of B Cell Lymphomas
Source: PLoS Biol. 2008 Jun 24;6(6):e152. doi: 10.1371/journal.pbio.0060152 (PMC2435152; doi:10.1371/journal.pbio.0060152)
Supplement: Table S1 — (37 KB DOC) [file pbio.0060152.st001.doc]

**Supplementary Table 1. Comparison of human and mouse tumors**

|  | **Latency to onset^1^** | **Aggressiveness** | **Anatomical presentation and evolution** | **Histopathology** | **Immuno-**  **phenotype** | *MYC* **over- expression** |
| --- | --- | --- | --- | --- | --- | --- |
| **Burkitt**  **Lymphoma** | 4-6 years of age | Very  aggressive | Jaw, lymph nodes, bone marrow, central nervous system, gastrointestinal tract | “Starry-sky”,  large lymphocytes with clumped chromatin and clear cytoplasm | Mature, activated B cells. | Yes, 8;14 and 8;22 translocations |
| **Eμ-*MYC*/BCRHEL/sHEL**  **transgenic mice** | 7 weeks | Very  aggressive | Spleen, lymph nodes, bone marrow, thymus and central nervous system **2** | “Starry-sky”,  large lymphocytes with clumped chromatin and clear cytoplasm **3** | Mature, activated B cells. | Yes,  Eμ-*MYC* transgene |
| **MMTV-rtTA/ TRE*MYC*/BCRHEL/sHEL transgenic mice** | 10 weeks | Very  Aggressive | Jaw, subsequent spreading to lymph nodes, spleen, bone marrow, liver, lungs**^2^** | “Starry-sky”,  large lymphocytes with clumped chromatin and clear cytoplasm **3** | Mature, activated B cells. | Yes,  MMTV-rtTA/TRE-*MYC* transgene |
| **B-CLL** | 61 years of age | Indolent, until  histological transformation | Spleen, subsequent spreading to lymph nodes, bone marrow, liver, lungs**^2^** | Small lymphocytes with clear chromatin and scant cytoplasm | Mature, Naïve, CD5+ B-cells | Yes, during progression, by gene amplification |
| **Eμ-*MYC*/BCRHEL**  **transgenic mice** | 18 weeks | Longer latency than two other mouse models described here | Spleen, subsequent spreading to lymph nodes, bone marrow, liver, lungs**^2^** | Small lymphocytes with clear chromatin and scant cytoplasm **4** | Mature, Naïve, CD5- B-cells | Yes,  Eμ-*MYC* transgene |

**1** **Mean age of highest incidence in humans [100, 101]. The latency for the murine tumors is defined as the median of the data in Figure 1.**

**2 Documentation of anatomical locations was determined by flow cytometric analysis for B220+/IgM- cells for
Eμ-*MYC* tumors, B220+/IgMa+ cells for both
Eμ-*MYC*/BCRHEL and Eμ-*MYC*/BCRHEL/sHEL tumors, and B220+/BCR^HEL^+ cells for MMTV-rtTA/TRE-*MYC*/BCRHEL/sHEL tumors. Anatomical distribution was confirmed by histological examination.**

**3 The tumors in the Eμ-*MYC* mice consisted of a mixed population of small cleaved and non-cleaved lymphoid cells, as well as larger centrocytes.**

**4 The tumors in the Eμ-*MYC*/BCRHEL mice consisted of a homogeneous population of lymphoid cells that appeared to be centrocytes.**
